# When Average Isn't Good Enough: Identifying Meaningful Subgroups in Clinical Data

**DOI:** 10.1007/s10608-023-10453-x

**Published:** 2024-01-28

**Authors:** Andrew T. Gloster, Matthias Nadler, Victoria Block, Elisa Haller, Julian Rubel, Charles Benoy, Jeanette Villanueva, Klaus Bader, Marc Walter, Undine Lang, Stefan G. Hofmann, Joseph Ciarrochi, Steven C. Hayes

**Affiliations:** 1 Division of Clinical Psychology and Intervention Science, Department of Psychology, University of Basel, Basel, Switzerland; 2 Center for Innovative Finance, University of Basel, Basel, Switzerland; 3 Psychiatric Hospital Sonnenhalde, Riehen, Switzerland; 4 Integrierte Psychiatrie Winterthur – Züricher Unterland, Winterthur, Switzerland; 5 School of Human Sciences, Clinical Psychology and Psychotherapy in Adulthood, Osnabrueck University, Osnabrueck, Germany; 6 Rehaklinik Centre Hospitalier Neuro-Psychiatrique Luxembourg (CHNP), Ettelbruck, Luxembourg; 7 University Psychiatric Clinics (UPK), University of Basel, Basel, Switzerland; 8 Psychiatric Center Wetzikon (Clienia Schlössli AG), Wetzikon, Switzerland; 9 Psychiatric Services Aargau (PDAG), Windisch, Switzerland; 10 Alexander von Humboldt Professor, Department of Clinical Psychology, Philipps-Universität Marburg, Marburg, Germany; 11 Institute for Positive Psychology and Education, Australian Catholic University, Sydney, Australia; 12 Department of Psychology, University of Nevada, Reno, USA

**Keywords:** Intraindividual differences, Idionomic analysis, Nomothetic, Processes

## Abstract

**Background:**

Clinical data are usually analyzed with the assumption that knowledge gathered from group averages applies to the individual. Doing so potentially obscures patients with meaningfully different trajectories of therapeutic change. Needed are “idionomic” methods that first examine idiographic patterns before nomothetic generalizations are made. The objective of this paper is to test whether such an idionomic method leads to different clinical conclusions.

**Methods:**

51 patients completed weekly process measures and symptom severity over a period of eight weeks. Change trajectories were analyzed using a nomothetic approach and an idiographic approach with bottom-up clustering of similar individuals. The outcome was patients’ well-being at post-treatment.

**Results:**

Individuals differed in the extent that underlying processes were linked to symptoms. Average trend lines did not represent the intraindividual changes well. The idionomic approach readily identified subgroups of patients that differentially predicted distal outcomes (well-being).

**Conclusions:**

Relying exclusively on average results may lead to an oversight of intraindividual pathways. Characterizing data first using idiographic approaches led to more refined conclusions, which is clinically useful, scientifically rigorous, and may help advance individualized psychotherapy approaches.

**Supplementary Information:**

The online version contains supplementary material available at 10.1007/s10608-023-10453-x.

## Introduction

Most studies in medical and behavioral science use statistics with the assumption that the conclusions derived from calculated mean-level results apply at least probabilistically to most members of the sample. Said in another way, it is assumed that measurement of central tendencies and variability between people can be used to model central tendencies and variability within persons.

Measurement theorists have long understood that this assumption could be wrong (Cattell, 1952) and it has sometimes been challenged in areas such as developmental psychology (Wohlwill, 1973), epigenetics (Gottlieb, 1992), or behavior analysis (Peters & Sidman, 1961). In the main, these intellectual objections have made little impact and most of the modern medical and behavioral science is based on normative statistical approaches.

Peter Molenaar (2004) appears to be the first to have linked this issue to a well-established concept in the physical sciences: ergodicity (Birkhoff, 1931; Boltzmann, 1885; von Neumann, 1932). Although its focus is on modeling the distribution of elements in space and time, in a more abstract sense the ergodic theorem describes the conditions under which data applying to a collective also applies to components of that collective. The theorem reveals that such an extension is only proper if the elements are ergodic. A simple way to think of ergodicity in a fashion that relates to medical and behavioral science is that for ergodic events the means and standard deviations of all individuals across time and for all cross-sectional samples of collections of individuals at any given point in time will be the same (Gates et al., in press). That is sometimes true and sometimes not in the physical sciences (Cherstvy et al., 2019; Galenko & Jou, 2019; Kuehn & Abrahamson, 2020), but it is rare to the point of being absent in the human and life sciences (Horst, 2008; Kelderman & Molenaar, 2007).

A small number of empirical studies have shown that nomothetic and idiographic analyses can lead to different conclusions (Fisher et al., 2018; Miller & van Horn, 2007; Molenaar & Campbell, 2009) but these ideas have not been widely adopted in intervention research. One reason may be that these studies have generally been based on complex methods or with data requirements that are far from typical research practices in the medical and behavioral sciences. For example, while complex network analyses, p factor analyses, replicated multivariate time series analyses, and other methods can be applied to longitudinal data sets when scores of within person observations are available, such data are usually not available from most randomized controlled trials, which typically have only a few measurement points. The net effect has been that theoretical alarms about the applicability of normative analyses have seemed empirically remote, and empirical tests of the need for idiographic data are both uncommon and readily set aside.

This issue has recently taken on special urgency in psychological intervention and the health sciences writ large because research attention is increasingly being focused on identifying processes of change that meaningfully and reliably apply to those presenting for treatment, in such areas as process-based therapy or personalized medicine. Focusing on processes of change creates a conundrum. On the one hand, a change process cannot be ergodic because stationarity is a key feature of ergodic events (Molenaar, 2013); on the other hand, knowledge drawn from a truly idiographic approach cut off from nomothetic conclusions cannot be applied to others. One possible solution would be for analyses to initially be based on idiographic conclusions, examined in the context of within person variability. These individual analyses could then be gathered into nomothetic generalizations that are ultimately shown to improve idiographic fit—what has been called an “idionomic” rather than normative approach (Hayes & Hofmann, 2021).

The absence of ergodicity does not invalidate nomothetic generalizations provided that generalizations are derived from intra-individual variation. It is important to examine ways to begin analysis entirely idiographically using commonly available data before gathering individuals into similar subpopulations and examining whether doing so improves the fit between individual behavior and subpopulation average behavior. Applied researchers may believe that they are doing something similar with moderation analysis, cluster analysis, growth curve modeling, longitudinal mixed regression models, and so on but these standard methods interpret individuals in the context of inter-individual variability first—precisely the step that may not be justified if the ergodicity assumption is violated (Molenaar, 2013). In contrast, if analyses that begin with intra-individual variability were routinely successful, intervention scientists might be able to select targeted interventions tailored to a more homogenous group or subgroup of similar patients. It remains empirically untested, however, whether such an approach would render more accurate and useful predictions in clinical trials.

Theoretically, subgroups can be based on any observable, treatment-relevant variable ranging from scores on a questionnaire, to genetic polymorphisms, to nurse observations. The growing evidence in support of a focus on processes of change, however, suggests that the treatment relevance of building subgroups will be increased to the degree that the grouping variable a) stipulates a hypothesized mechanism of action that can be assessed and manipulated, and b) predicts a clinically relevant outcome; e.g., visual acuity, white blood cell count, mortality, depression symptoms, well-being, and so on (Gloster & Karekla, 2020; Hofmann & Hayes, 2019).

The present study implemented this process-based strategy with a sample of patients who were assessed on hypothesized mechanisms of action consistent with state-of-the-art theories of change during psychotherapy (Gloster et al., 2017; Hayes et al., 2019; Kashdan & Rottenberg, 2010) in addition to the measurement of typical syndromal features. We included variables previously shown to be active mechanisms of change in psychotherapy using nomothetic methods, such as mindfulness, relating differently to one’s thinking, and acting on one’s values (Hayes et al., 2020).

Different patients will respond differently to exercises designed to change such processes: e.g., some will improve their mindfulness skills, others may not change at all or even worsen in their ability to be mindful. Because change occurs over time, it is necessary to collect longitudinal data to model change trajectories (Gloster et al., 2014) and to assess it in terms of intra-individual variability. In the long run, daily diary apps or wearable automated data collection may allow the routine use of longitudinal models that are based on a series of several dozen longitudinal data points (Gates & Molenaar, 2012; Gates et al., 2017), but at the moment, data of that kind is rarely part of existing research designs. In the present study, we examined the application of a relatively simple idiographic approach built upon a limited number of data points of the kind routinely collected in clinical practice. In this approach, each individual was characterized in a bottom-up way that involved estimating individual effects that were uninfluenced by other individual effects. These were then clustered in a fashion that never treated the individual as “error”.

In the present study, this idionomic strategy was used to examine three key aims: (1) test whether assumptions of ergodicity are met or violated in clinical data; (2) test whether building subgroups using a bottom-up idiographic procedure before nomothetic analysis leads to different conclusions about underlying change than traditional nomothetic approaches; and (3) test whether idiographically identified bottom-up subgroups predicted a clinically meaningful distal outcome (namely, well-being), suggesting possible advantages for a more idionomic approach. These questions were examined using longitudinal data from a real clinical sample with time series data. Examining these questions under real world conditions was designed to maximize the chance that the results are clinically relevant.

## Methods

### Participants

All methods were approved by the local ethics committee and all participants completed an informed consent. Data were derived from the “Choose Change” transdiagnostic controlled effectiveness clinical trial focused on treating non-responding patients diagnosed with a DSM-IV disorder in an inpatient setting using Acceptance and Commitment Therapy (ACT), a common transdiagnostic intervention method (Gloster et al., 2023, 2020a, 2020b; Villanueva et al, 2019). Participants for this study were a subgroup of the 108 in-patients; namely those who completed all weekly assessments during active treatment from week 1–8 without gaps or missing items (n = 51). Additionally, as treatment usually lasted 8–12 weeks, two patients who stayed in treatment longer than 14 weeks were removed as outliers for these analyses. Table 1 presents detailed sample statistics.

**Table 1:** Sample characteristics

	Total N = 51
Age	
Mean (SD)	33.61 (10.14)
Sex	
Females N (%)	22 (41.51)
Diagnoses N (%)	
Mood disorder	30 (58.82)
Anxiety disorder	39 (76.47)
Obsessive compulsive disorder	20 (39.22)
Other disorder	20 (39.22)
Two or more diagnoses	36 (70.59)

### Assessments

Assessments included six process variables, one proximal outcome variable (i.e., weekly assessed symptoms) and one distal outcome variable (i.e., participants’ ratings of well-being at post-treatment). The process variables were completed weekly using the Psy-Flex (Gloster et al., 2021). The Psy-Flex measures skills of psychological flexibility and is sensitive to change produced by the intervention used in the original study. The items query about: (1) being present (“Even if I am somewhere else with my thoughts, I can focus on what's going on in important moments”), (2) acceptance (“If need be, I can let unpleasant thoughts and experiences happen without having to get rid of them immediately”), (3) defusion (“I can look at hindering thoughts from a distance without letting them control me”), (4) steady self (“Even if thoughts and experiences are confusing me I can notice something like a steady core inside of me”), (5) own values (“I engage thoroughly in things that are important, useful, or meaningful to me”), and (6) being engaged (“I determine what's important for me and decide what I want to use my energy for”). Weekly reports of the level of suffering caused by symptoms as assessed with one self-constructed item served as the proximal outcome variable (“During the last seven days, how much did you suffer because of your symptoms?”). When completing weekly assessments, participants reported on their experiences for the previous seven days. The distal outcome variable measured patients’ subjective level of well-being as assessed by the Mental Health Continuum—Short Form (MHC-SF) and was collected pretreatment and post-treatment (Keyes et al., 2008).

### Nomothetic Analytical Approach

To test the nomothetic approach, we calculated the aggregated interindividual measurements for each variable at the group level (i.e., grouping all participants together for statistical analysis). The following statistics were calculated: Item mean change and standard deviations were calculated for each individual and then aggregated (1) per item per time point (i.e., week); (2) weekly mean change per item; and (3) baseline to posttreatment change by calculating the difference of week 1 and week 8 per item. A linear model was created for each item estimating a linear trend (slope and R-squared of the linear model). For better interpretation, the linear models for each item were visualized.

### Test of the Ergodic Assumption

Ergodicity was formally examined based on the extent to which relationships between the processes and symptoms significantly varied within a person. Multi-level analyses were calculated using the lme4 package (Bates et al., 2015), nesting all observations within person. For every link between process and outcome, we compared a multi-level model that assumed random intercepts (allowing people to differ on the dependent variable) with one that assumed both random intercepts and slopes. A significant difference indicates that slopes between process and outcome differed between people, or that the within-person relationship between process and outcome differed from person to person.

### Idionomic Analytical Approach

In order to examine change using an idionomic strategy, we first examined each individual’s change trajectory over the eight weeks of observations. These individual change trajectories were then used to determine whether there were groups of individuals that had similar trajectories using cluster analysis to arrive at these nomothetic sub-populations. No averages across individuals or traditional estimates of inter-individual variation were built into the clustering processes used.

To conduct this analysis, we first calculated trend/regression lines for each individual and item separately. Therefore, no error term was calculated at the group level. Intra-individual trends are a discrete aspect of intra-individual variation, but it should be noted that intra-individual error terms can be also calculated for each individual. For example, intra-individual error terms can assess the degree to which the rate of change (slope) was not equidistant between time points for that participant. In the present study, such error terms factored into the analytic steps only in our assessment of ergodicity (e.g., Fig. 4 below).

Because we were ultimately interested in identifying meaningful clinical groups, we considered both an individual’s baseline value and their slope of change in subsequent steps. In order to facilitate weighting and interpretation, raw values (i.e., 1–5) were transformed so that values would be between 0 and 1 for the intercept and slope. This transformation did not alter the actual distance between individual’s trend lines and no error term was used. To achieve this, we construct a normalized weighted distance based on the intercept and slope of the linear model for each participants’ time series:

$$d\left(x,y\right)=\left|\alpha *(norm\left({x}_{intercept}\right)-norm\left({y}_{intercept}\right))\right|+|\beta *(norm\left({x}_{slope}\right)-norm\left({y}_{slope}\right))|$$

The weights for the intercept and slope are $\alpha$ and $\beta$ respectively and normalization transforms the values for intercept and slopes to values between 0 and 1 across all samples (i.e., the highest intercept is 1 and the lowest intercept is 0).

#### Choosing a Distance Measure

Clustering observations (i.e., individuals) requires a distance measure to determine the similarity of observations. Our observations are short time series and there are different ways to calculate the similarity of two time series. In the absence of a universal method to compare time series and because the choice of distance measure has an impact on the result of the analysis, it is important to be very clear about the properties of the observations that were considered.

For our analysis, we needed a distance measure that captured two key metrics: The initial magnitude of an item (severity level) and the trend the item follows over the eight weeks (change). The latter was given more importance than the first a priori in this manuscript, in part because the intercept influences the trajectory in the form of ceiling/basement effects and regression to the norm (i.e., a high intercept leaves little or no room for gains, low intercept increases the likelihood of gains).

#### Choosing Parameters for the Clustering

When choosing a distance measure, one can think of each individual’s multiple observations over time as a single observation, having a location based on its regression parameters. The distance between two such observations is then given as the distance between their respective locations. To calculate the distance between two clusters, all the observations in both clusters have to be considered according to a linkage criterion. Our goal was to choose the linkage criterion resulting in the most robust and well-formed clustering structure given our data and our clustering algorithm. From the commonly used linkage criteria—single, complete, average, centroid, and Ward’s (Jarman, 2020)—that we tested, Ward’s minimum variance method (Ward, 1963) consistently resulted in the highest agglomerative coefficient (AC) by minimizing the total within-cluster variance. For our chosen clustering method, Agglomerative Nesting (AGNES) clustering, the clustering structure can be measured with the AC. The AC reveals the mean of the normalized lengths at which clusters are formed, and higher values for the AC indicate a stronger clustering structure (Gates et al., 2017).

When choosing the parameters to measure distance measures, weights to the *α*- and *β*-coefficient must be assigned. As we wanted to compare linear regression lines with each other, we needed to assign the parameters for intercept and slope, respectively. For reasons explained above, we assigned the slope more weight than the intercept, so that linear regressions with similar slopes were more likely to be grouped together as long as their intercepts are not too different. This is a practical way of being able to compare individual trajectories.

For the parameters $\alpha$ and $\beta$, we found that the values $\alpha =1$ and $\beta =3$ separated our time series in a meaningful way. These numbers were obtained by testing different values for $\alpha \mathrm{ and }\beta$ and comparing the resultant figures via visual inspection for which slope and intercept had the largest impact on the categorization (see supplementary material section A for examples). When participants showed similar changes over time, they were more likely to be grouped in a cluster by the function. When defining the parameters for distance, we used clustering methods that employed the Manhattan distance measure (Gonzalez & Palais, 1962), which measured the actual distance traveled between points (around the corners), and not the Euclidian distance (direct).

#### Choosing a Clustering Approach

In order to determine groups of individuals with similar change patterns we used clustering procedures. Clustering procedures must satisfy at least the following properties described by Xu and Tian (2015):

1. Instances in the same cluster must be as similar as possible
2. Instances in different clusters must be as different as possible
3. Measurements for similarity and dissimilarity must be clear and pragmatic

Based on these axioms, there are different methods that can be used to make clustering decisions. Approaches that start with each observation as its own cluster are commonly referred to as Hierarchical Clustering. To choose a metric, we ran our data through the following clustering sequences: AGNES and Divisive Clustering (DIANA). The outcome of the two approaches were compared to choose the more appropriate method, as literature has not yet defined which is preferable in all instances (Edelbrock & McLaughlin, 1980). DIANA is a top-down method of hierarchical clustering that uses a reversed algorithm, starting with the assumption that all data points belong to the same cluster. In each iteration, the largest available cluster is then split into two clusters until each observation represents one cluster. The algorithm therefore needs n-1 iterations to complete. How clusters are split depends on how the parameters of dissimilarity are defined. AGNES, conversely, is a bottom-up approach commonly used to find and group similar observations in a set of data. It creates clusters in an iterative approach, starting out with as many clusters as there are observations, and with each step combining those that are most similar to each other to form a bigger cluster. In each iteration, the most similar clusters are combined until in the last step, all observations are merged into one cluster. Generally, AGNES yields more balanced clusters than DIANA. So far, there is no established way of deciding the best algorithm, which is why one has to define and justify the chosen parameters carefully. The results are then evaluated by comparing the cluster sizes on the one hand and visually checking the groups’ trajectories on the other hand. Comparing the DIANA and AGNES clustering algorithms showed that the AGNES algorithm yielded more balanced clusters for our data (for an example of DIANA clustering, see Supplements B and C). Hence, we used AGNES for the idiographic clustering. To interpret the result of the clustering visually, Dendrograms, graphs that show the iteration like the root-system of a tree, were inspected. It is important to note that depending on the data and the clustering parameters, the most fitting clustering algorithm may differ.

#### Choosing the Number of Clusters

A four-cluster solution was chosen on the assumption that there are four possible therapy outcomes: slight and strong increase of parameters, and slight and strong decrease of parameters. It would also be possible to base the number of clusters on a statistic measure like the silhouette plot, but there are no objective criteria for the correct number of clusters to choose.

#### Prediction of Distal Outcome

In order to examine whether the idiographically derived clusters of patients predicted clinically useful information nomothetically, we subsequently tested whether the clusters differed with respect to a distal outcome. Specifically, we tested whether patients in the clusters subsequently resulted in meaningfully different values on well-being. This was implemented by adding each patient to one of four factor levels based on their AGNES-derived groups for each process variable and then testing differences using an omnibus ANOVA. As the sample sizes of the resulting clusters were underpowered for such analyses, we concentrated on effect sizes using Cohen’s f, an effect size measure used for ANOVA using pooled standard deviations.

## Results

### Nomothetic Approach

Results (see Table 2) showed that, on average, the *seven observed items* either revealed slopes indicative of some improvement over time, or no change. The linear models per item showed varying slopes, with some regression lines’ slope being close to zero (e.g., item 1—Being present; item 6—Being engaged;), suggesting that on average, no change had happened over the eight weeks that were measured. The weekly mean difference per item was limited for all items, with the highest score accounting for a 2% change over one week on the Psy-Flex scale (from 1–5). R-squared values per item ranged from 0.02 to 0.74. As r-squared values are lower, the poor fit of a purely linear nomothetic model indicates that there may be patterns in the data that are being overlooked due to the aggregating nature of nomothetic analysis.

**Table 2:** Nomothetic changes and results of linear models (N = 51)

	Weekly mean difference	Pre-post mean	Standard deviation	rmssd	slope	r-squared
1. Being present	− 0.006	− 0.039	0.84	0.84	− 0.006	0.020
2. Acceptance	0.081	0.569	0.93	0.94	0.057	0.712
3. Defusion	0.090	0.627	0.96	0.98	0.057	0.525
4. Steady self	0.084	0.588	1.07	0.96	0.066	0.738
5. Own values	0.076	0.529	0.83	0.87	0.049	0.505
6. Being engaged	0.017	0.118	0.81	0.75	0.009	0.229
7. Symptoms	− 0.036	− 0.036	1.10	0.95	− 0.022	0.480

Weekly mean differences calculated across participants and between weeks. Pre-post mean calculated by subtracting overall mean value of week 8 from overall mean value of week 1. Standard deviation (SD) reflects inter-individual SD across all weeks. Rmssd, slope, and r-squared calculated by setting up a linear model

**Fig. 1:**
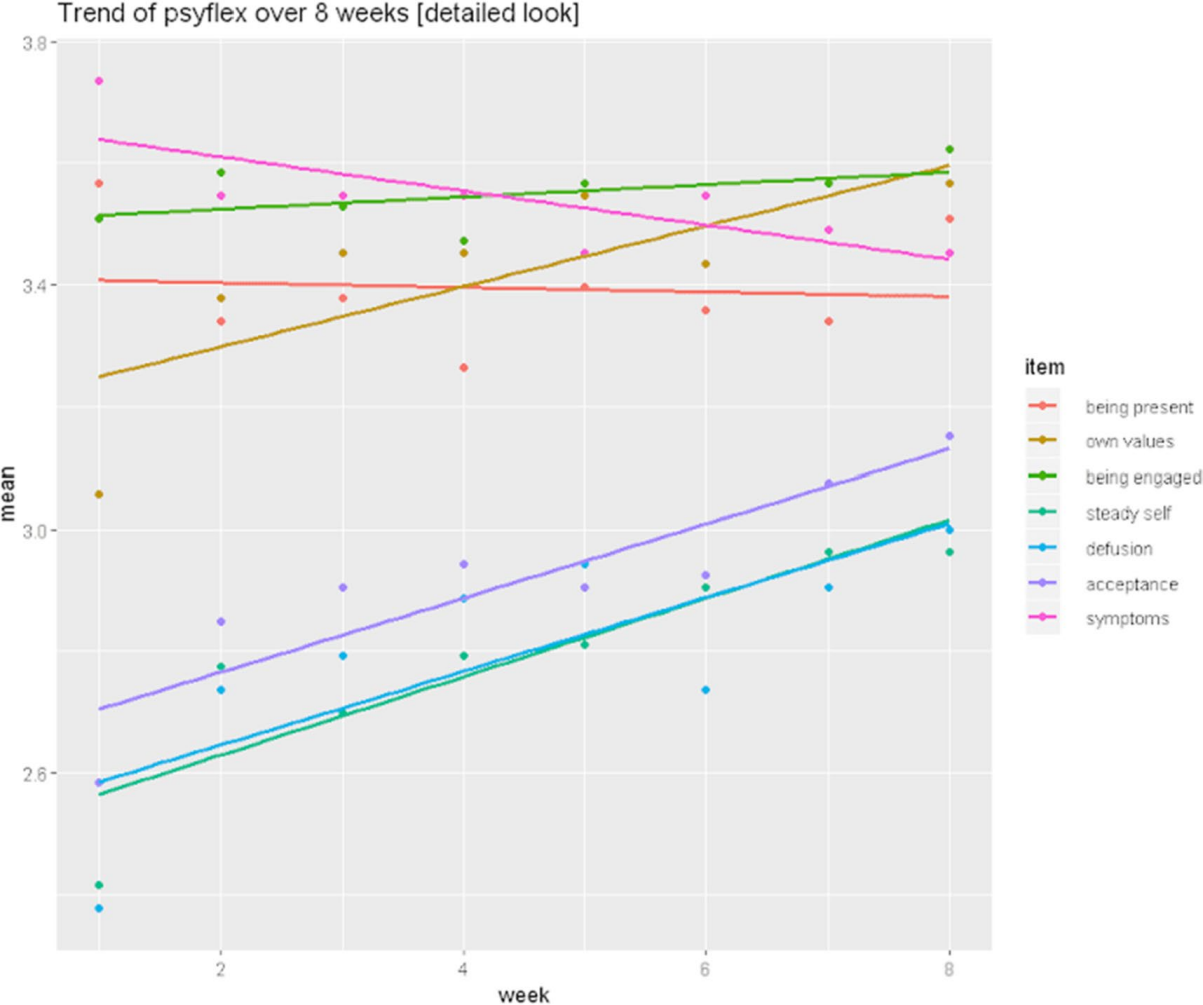
Average trend lines for items measuring psychological flexibility and symptoms

Figure 1 shows the weekly means and the linear model for the items plotted over eight weeks. On average, *symptoms* shows a negative trend, the processes of *being present* and *being engaged* show no significant trends, and the remaining process items (*acceptance*, *defusion*, *own values*, and *steady self*) show positive trends.

**Fig. 2:**
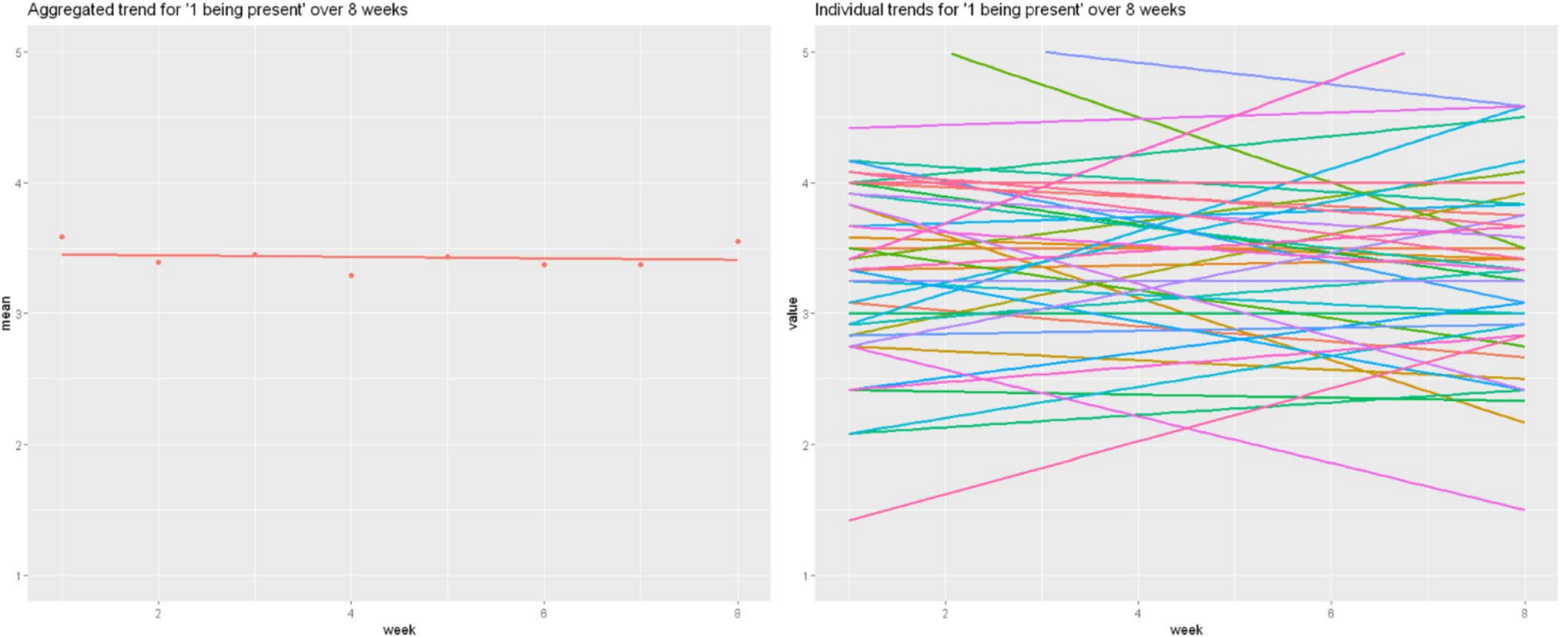
Individual regression lines vs. aggregated regression line for the item “Being present”

Often, time-series data is analyzed using multi-level models, because such data tend to contain both missing data and an unequal number of observations (Snijders & Bosker, 2012), which is not the case here. Multi-level models consider inter-individual variance as a metric for group level estimates, however they make the assumption of normally distributed individual trajectories around the average intercept and slope, resulting in a restrictive model. To exemplify this, consider the first item, *being present*. As seen in the left side of Fig. 2, the mean group level change for this item showed that minimal change across time occurred. The right side of Fig. 2 shows an individual regression line for each participant in the sample (trends instead of point measures are displayed for better readability of the plot). A very different picture emerges when all 51 of the individual trend lines are contrasted with the aggregated trend line.

The nomothetic measurement not only fails to describe the individual trends, but it is actively misleading in that it suggests that there is very little change over time. For many individuals that is hardly the case. The observed inter-individual standard deviation (see Table 2) hints that there is more going on, but standard deviation alone does not consider that each trend is a time series, nor the degree of intra-individual variability.

**Fig. 3:**
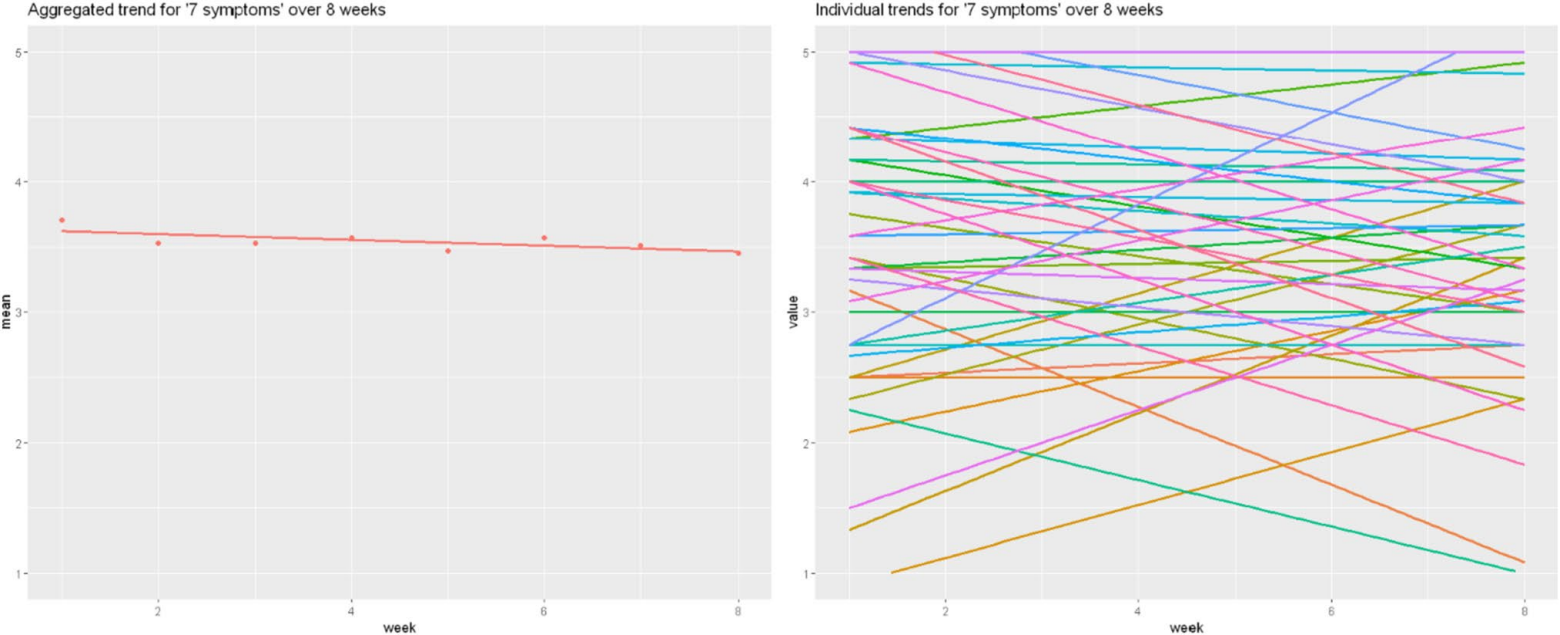
Individual regression lines vs. aggregated regression line for the item "Symptoms"

In order to examine the generalizability of this pattern, Fig. 3 repeats this analysis with the item measuring symptoms. The same pattern can be seen. Indeed, if we were to continue item by item, it is universally true that the nomothetic pattern fails to characterize most of the sample. For the aggregated and individual trend lines for the remaining items, please see supplementary section D.

### Test of Ergodic Assumption

If we assume ergodicity, then the link between process and symptoms should not significantly differ between people. We evaluated this possibility using multi-level models that estimated intercepts and betas (relationships between process and symptoms) for each person. In Table 3, we present the fixed effect (average), and the 10th and 90th percentile of the distribution of participant betas. The negative fixed effects indicate that all weekly processes were generally associated with lower symptoms, though the fixed effect for *own values* was the only process to reach traditional statistical significance. However, the Chi-square tests of varying slopes were all significant, indicating that the relationship between the weekly process and weekly symptoms significantly varied from person to person. This within person variance appeared to be largest for *defusion* and *steady self*. Although processes were generally beneficial (negative fixed effects), there was a subset of people for whom the processes were either inert or possibly associated with worse symptoms, especially for *steady self* (upper beta = 0.28) and *defusion* (upper beta = 0.24).

**Table 3:** Betas and range of within person associations between processes and negative symptoms

Process	10%	Fixed effect	90%	Chisq
Being present	− 0.32	− 0.07	0.07	15.08***
Acceptance	− 0.33	− 0.12	0.14	10.03**
Defusion	− 0.44	− 0.12	0.24	38.5***
Steady self	− 0.37	− 0.12	0.28	25.65***
Own values	− 0.28	− 0.13*	0.11	16.44***
Being engaged	− 0.24	− 0.09	0.14	14.22***

Chi-square tests that the relationship between process and symptoms varied by participant

*p < 0.05, **p < 0.01, ***p < 0.001

**Fig. 4:**
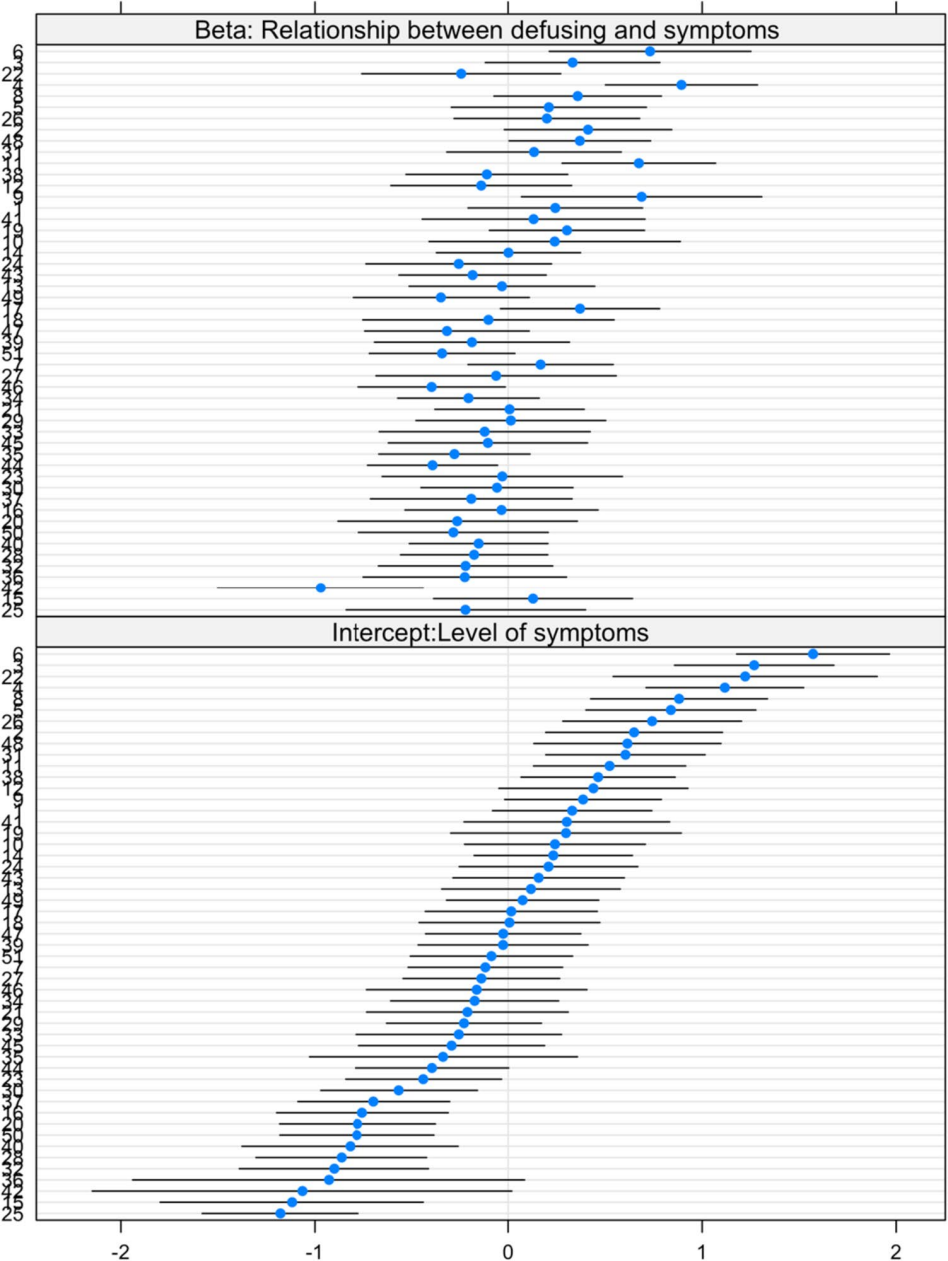
Standardized scores for the intercept and within person relationship between weekly defusing and weekly symptoms

As an example of the implications of a lack of ergodicity, the within-person relationship between defusion and symptoms is illustrated in Fig. 4. We can see that for some people, higher defusion is significantly associated with worse symptoms (e.g., see participant 6 and 22, for whom intra-individual standard error bars do not overlap with zero). For others (e.g., participant 42 and 44), defusion is associated with fewer symptoms.

### Idionomic Approach

**Fig. 5:**
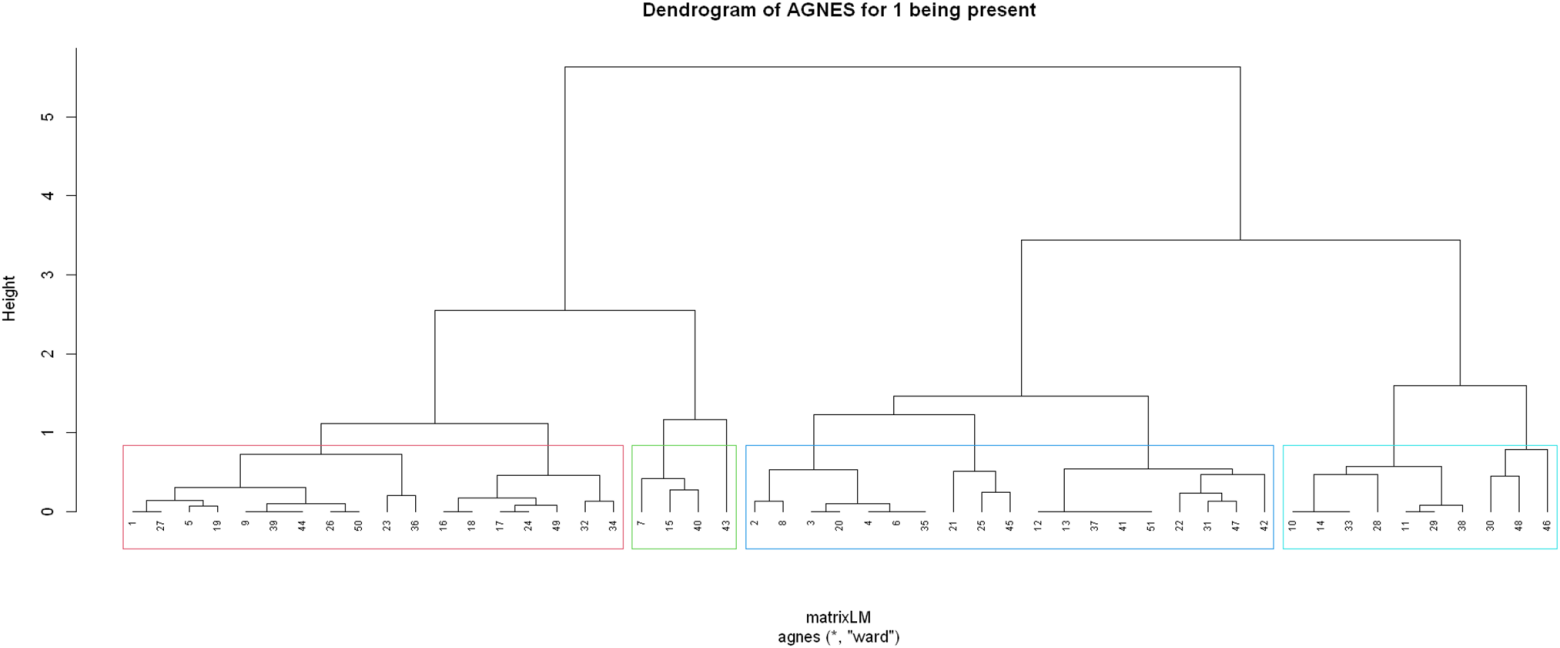
Dendrogram for Item 1 (being present) using the AGNES clustering method

In order to determine whether there were underlying subgroups of change trajectories, data were submitted to a clustering procedure. The results of the clustering are shown for the first item *being present* but we ran the analyses for all of the other items as well (see supplementary section B for details). For the item *being present*, the hierarchical clustering using AGNES is shown in Fig. 5 (see the clusters for the remaining items in supplementary section D). The Dendrogram demonstrates the four-cluster solution, which was chosen for theoretical reasons (see methods section). The height of the vertical lines is a measure of similarity. The shorter the line, the more similar the observations within the clusters. AGNES used the $\alpha$- and $\beta$- weights as defined above and joined the most similar two clusters in each iteration. For the above-mentioned theoretical reasons, the AGNES clustering sequence was stopped at a solution with four clusters.

**Fig. 6:**
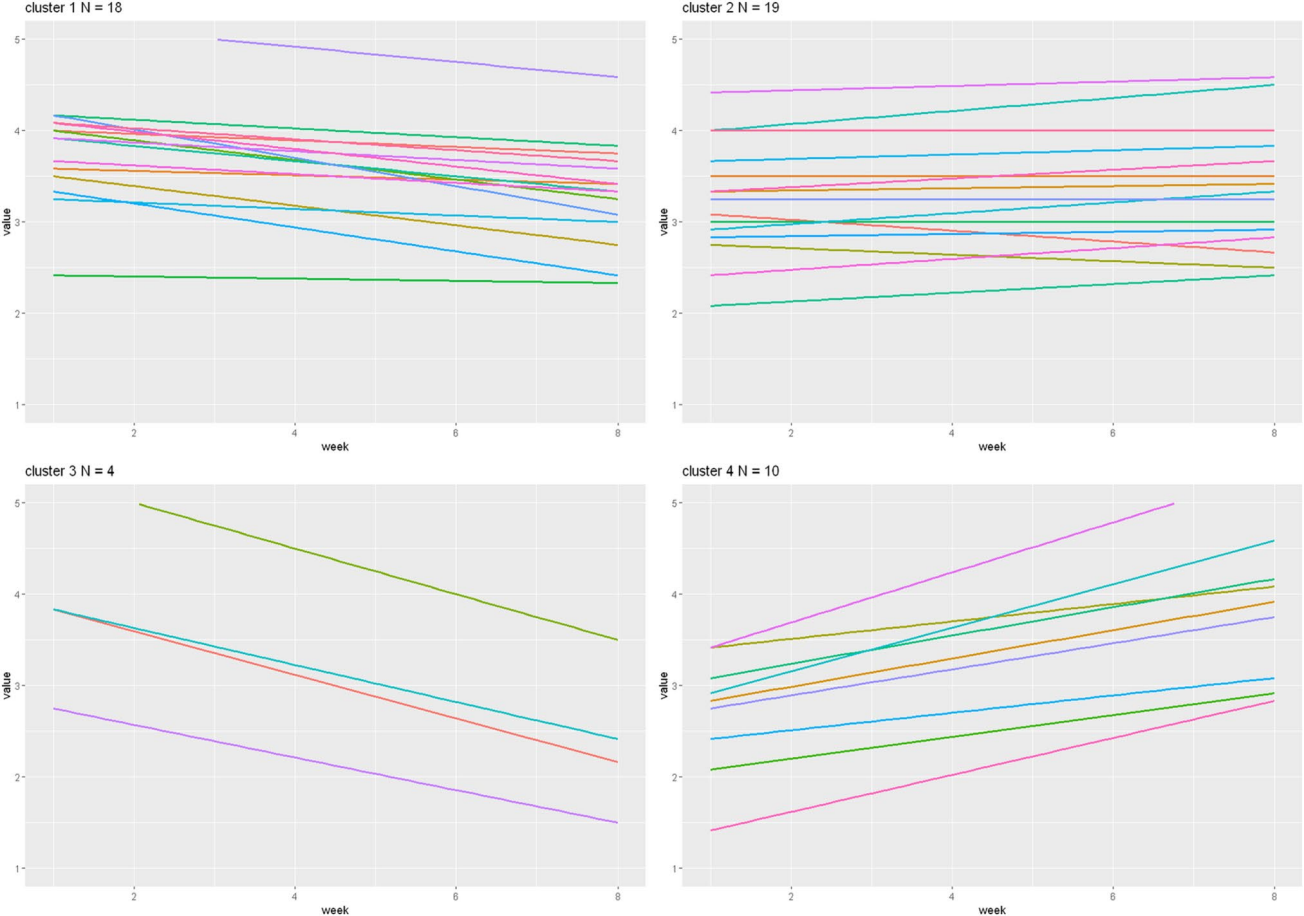
Clusters of Item 1 (being present) with AGNES method

To better understand the outcome of the clustering sequence, we visualized the participants’ individual trend lines for *being present* grouped by the resultant four clusters (see Fig. 6). The algorithm clearly divided the data into similar groups, based first on the slope (with a weight of 3) and second on the intercept (with a weight of 1). Each cluster shows a distinctive trend: patients of cluster 1 show a declining trend over the course of eight weeks, patients of cluster 2 reported little positive or no change, patients in cluster 3 reported a large decline over the course of therapy, and patients in cluster 4 experienced a large gain in *being present*. All the other variables also produced four cluster solutions, with similar characteristics regarding their trajectories (see supplemental material section E).

**Fig. 7:**
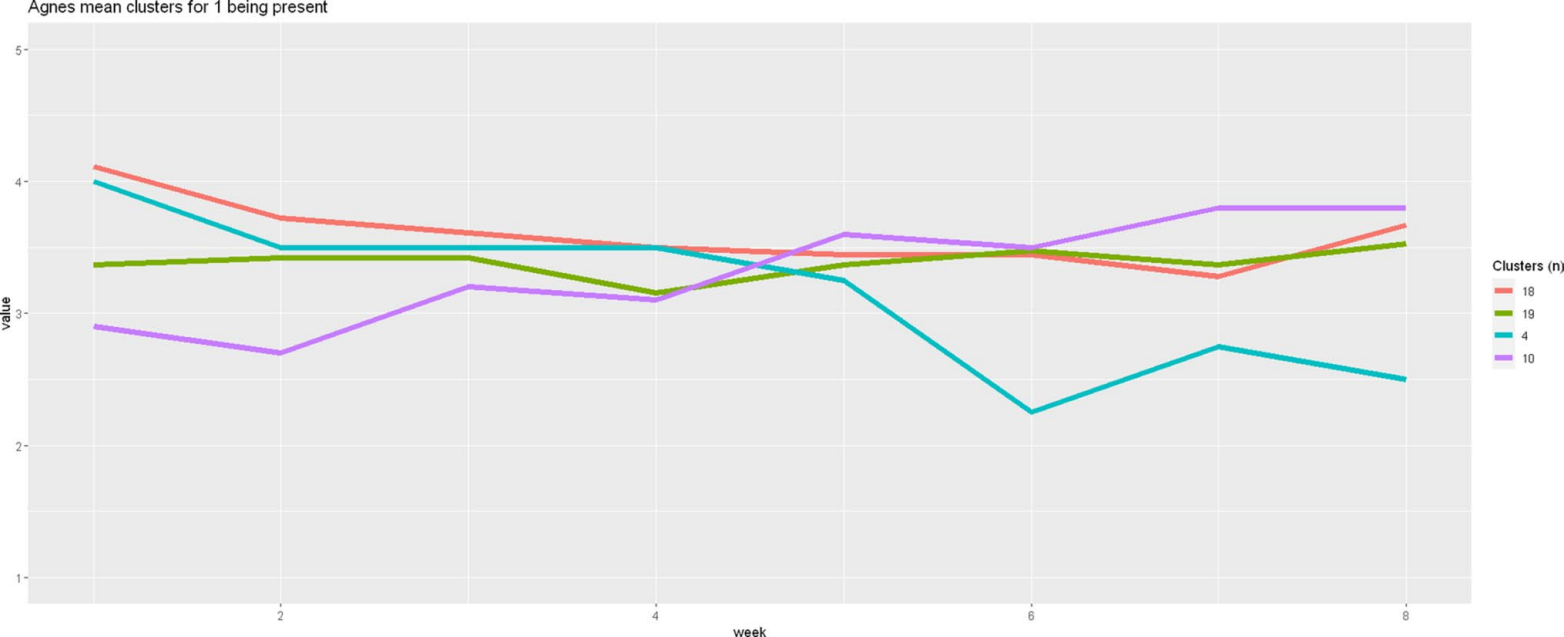
Average cluster time series for Item 1 (being present)

After identifying these subgroups of change, we then (as a last step instead of a first step) averaged the time series at each time point for the patients within each cluster for each variable. Figure 7 shows the data for *being present* as an example. As is clearly visible, the aggregated cluster changes differ from the average mean line. It becomes apparent that one of the largest groups (N = 18) shows a slow decline with a slight gain between week 7 and 8. In contrast, there is a group (N = 10) that reports continuously rising values, one small group (N = 4) that reports overall declining values, and another large group (N = 19) that reports no significant changes. Theoretical interpretation of this finding aside, the figure shows very clearly that different courses exist for this variable and that clustering the sample in a meaningful way leads to interpretable results, where a single average line, where all data is collapsed together, does not. For examples of the clustering results for items 2 to 7, please refer to supplemental material section F.

### Nomothetic Prediction of Distal Outcome

In order to examine the clinical utility of these clusters, we tested whether the derived clusters per each hypothesized process items differed with respect to the distal outcome variable of well-being assessed separately at post-treatment. As expected, the omnibus ANOVA was not significant, likely because of the small sample size and the observation above that clusters cancel each other out (some patients increase, and others decrease). Based on this we examined the planned contrasts in order to understand the clinical implications of the derived clusters. Within the idionomically derived groups for *own values,* the increasing well-being scores of individuals from group 2 (relatively no change in *own values*, *p* = 0.031), group 3 (continuous gains in *own values*, *p* = 0.012), and group 4 (early gains in *own values*, *p* = 0.047) differed from group 1 (decrease in *own values*), with an overall large effect size (Cohen’s *f* = 0.41). For *defusion*, the changes in well-being scores of group 4 (continuous gains in *defusion*, *p* = 0.48) differed from group 1 (decrease in *defusion*), resulting in a medium effect size (Cohen’s *f* = *0.34*). For *acceptance,* group 3 (continuous gains in *acceptance*, *p* = 0.042) and group 4 (no change in high baseline of *acceptance*, *p* = 0.042) differed from group 1 (decrease in *acceptance*), reaching a medium effect size (Cohen’s *f* = 0.37). Cohen’s *f* therefore differentially predicted well-being with respect to the process variables of *own values, defusion,* and *acceptance.* Although visual inspection of the graphs showed differences across the clusters, the remaining process variables showed only low effects with respect to the difference in outcomes of well-being: [*being Present* (*f* = 0.19), *being engaged* (*f* = 0.13), *steady self* (*f* = 0.13)]. Idionomically derived clusters based on the intermediate outcome variable of *symptoms* (*f* = 0.26) also did not result in significant differences with respect to the distal outcome of well-being, with a medium effect size. See supplementary material in section G and H for more details.

## Discussion

The efficacy of psychotherapy for mental disorders is well documented. The vast majority of studies show that on average patients improve to a statistically significant degree and that this change is clinically meaningful (Butler et al., 2006; Gloster et al., 2020a; Gloster et al., 2020b; S. Hofmann et al., 2010). Although it is an oversimplification to assume that results from randomized trials apply to a given individual, this interpretation is common and individual patient trajectories are extended from such studies into clinical care. The present study presented a method for identifying different patterns of change and illustrated that meaningful subgroups can be found even when the intervention has been shown to be effective at the group level. The present study also points towards one possible way to methodologically and empirically explore such sub-groups.

Ergodicity requires both stationarity and that the same dynamic model applies to all constituent elements. In the present study, the data did not meet the ergodic assumption. We found that, for example, defusion was related with either more symptoms, less symptoms, or not related at all—depending on the individual. Furthermore, very different trajectories were present over a treatment of eight weeks. Cluster analysis based on idiographic data identified distinguishable patterns of deterioration, no change, or improvement. When looking at week-by-week patterns, a group of sudden gains was visible. When results were examined idiographically relevant to intra-individual variability, the relationship of a given process to outcome could vary from significantly positive to significantly negative. Thus, both of the key requirements for ergodicity (stationarity and common dynamic models) were violated. Because the ergodic assumption was demonstrably violated in these clinical data, relying exclusively on nomothetic results would misrepresent some individuals. It is not hard to see why from the present data: at the purely nomothetic level these idiographic differences largely cancelled each other out, and process to outcome relationships were no longer evident.

Recent studies on ergodicity have reached the same conclusion, though the percentage of a sample that belongs to a sub-group that is not characterized by the nomothetic finding differs (Fisher et al., 2018; Sahdra et al., 2023). The present results also suggest one possible way forward. In the present approach, analyses began by examining entirely idiographic data linked to intra-individual variability. Nomothetic clustering procedures that avoided inter-participant averaging as input then identified multiple sub-groups of patients with varied patterns of change. These bottom-up analyses allowed for the identification of differentiated patterns person by person that predicted a clinically useful distal outcome, well-being, for at least some of the key processes (acceptance, defusion, and own values) that were targeted in the underlying psychotherapy trial. Conversely, the three other targeted processes (being present, steady self, and being engaged) and idionomically identified patterns of symptom change, were not related to distally assessed well-being. Said simply, bottom-up idionomic analyses lead to different conclusions than top-down nomothetic analyses. It is not widely appreciated that purely nomothetic conclusions and those based on idiographic data within the same population can be virtual opposites. This is known as *Simpson’s paradox* and it is quite common in behavioral and medical areas (Kievit et al., 2013). As a simple example, in all large groups of people better typists will both type fast and make fewer errors per typed word, resulting in a negative correlation between speed and errors; nevertheless, as individuals, every single typist regardless of expertise will make more errors the faster they type, reversing that relation. Keivit et al. (2013) suggest several steps to avoid falling prey to Simpson’s paradox, including several of the steps taken here: intervene; study change over time idiographically; conduct cluster analysis; and visualize the data.

In order to make progress towards personalized interventions, it is important to understand multiple patterns of change in their own right. By extension, this will help advance our understanding of the underlying processes of change initiated by an intervention. The exact number of subgroups and trajectories is likely a function of the subject matter, intervention, and clinical presentation of patients. Although these patterns shown here are largely consistent across the variables examined in this study and with patterns hypothesized in the literature (Lutz et al., 2013), we do not claim that these are the only patterns that exist. Indeed, much more research is needed to help identify the nature of change itself. We do argue, however, that the idionomic results from the present study can be extended to individuals if idiographic patterns are the basis for such an extension.

In the present study with patients suffering from mental disorders, the idionomically derived clusters of change based on accepting and defusing from one’s thoughts as well as engaging in values were most predictive of the distal outcome of well-being. Clusters of change patterns based on other variables were less differentially predictive of the distal outcome. Importantly, across all process variables and symptoms, the trajectory of the cluster of patients that declined the most by the end of treatment was already visually discernible from the other cluster of patients by the fourth or fifth week in the graphs depicting week-by-week change per cluster. In a fully idionomic approach, the implications of such nomothetic extensions would be formally tested. For example, when treating patients suffering from mental health issues a clinician might pay particular attention to the weekly development of acceptance, defusion and values after a month or so of treatment. If the patterns observed here are replicated in other studies, it would enable clinicians to begin to pinpoint the timepoint within an ongoing psychotherapy at which their current patients’ outcome is most predictive of an outcome similar to the bottom-up derived empirical clusters. If one’s current patient’s course is most identified with a cluster associated with high levels on the desired distal outcome, then the therapist can hold the course. If, however, the patient’s course is most similar to a cluster associated with poor prognosis, changes can be introduced. Such individualization of the treatment would save resources and hopefully improve the lives of patients via more efficient care.

It is noteworthy that these results were obtained in a real-world clinical sample that was relatively small in terms of participants and time points. The bottom-up idionomic approach thus appears to be reasonably sensitive, perhaps in part because the building of homogeneous subgroups partitions the variance in ways that increase statistical power. This could be an advantage for clinical research aimed at understanding processes of change.

Other lines of research on the science of change suggest that there is fertile ground for idionomic approaches. Personalized medicine, for instance, aims to tailor interventions by grouping patients into categories based on indicators that are predictive of outcome. Within the field of psychotherapy, efforts are underway to systematically organize the understanding of processes of therapeutic change as something separate from the techniques or therapies that give rise to change. Examining the processes of change using idionomic bottom-up approach may help realize this goal.

This study has multiple limitations. First, our sample size was relatively small. Larger sample sizes will allow us to detect more complex patterns of change and will be more representative of the clusters we are likely to identify in the population. Second, the iterative process used in this study of calculating the clusters separately for each variable does not allow for the real possibility that the variables dynamically influence each other’s development. We did not have sufficient power to conduct such dynamic analysis. To our knowledge, such multivariate approaches are currently missing and statistical work in this area is needed. Third, although this approach allowed for clustering of shorter time series than the currently most used models, a good approach to model individual cluster time-series analysis for shorter time-series is also needed. Likewise, the idionomic approach used here relied on distance between observations to derive clusters. Approaches are needed that can also use time as a weighted parameter. Fourth, for clarity and simplicity we included participants with complete data sets. Future research may examine what effect missing values have on the results. Finally, these real-world data were derived using one intervention (i.e., Acceptance and Commitment Therapy) and the variables targeted the hypothesized processes of change. It is necessary to test whether these or other clusters of change emerge with different therapies and targeted processes of change.

To rectify these limitations, future studies should focus on systematically investigating how to translate results from this approach into clinical practice, comparing them to other emerging idionomic approaches (Gloster & Karekla, 2020; Snijders & Bosker, 2012). This could include further tests of what distal outcomes the idionomically identified clusters predict and which they do not. Studies could also explicitly test whether tailoring treatment based on clusters improves outcomes for individual patients.

In conclusion, as clinical change applies to particular people, average is not enough. Whereas nomothetic approaches inform about the efficacy of an intervention in general, ergodicity is seldom present and the nomothetic statistics are incomplete and may at times lead to faulty conclusions. Additional, bottom-up idionomic approaches are needed to augment traditional knowledge from clinical trials to better understand the process of change and to use this knowledge to provide better treatments for all subgroups of patients.

### Supplementary Information

Below is the link to the electronic supplementary material.

Supplementary file1 (DOCX 957 kb)

## Data Availability

The datasets generated during and/or analyzed during the current study are available on OSF, https://osf.io/a9hqe/?view_only=21f089f69fec4417bbd1cc4b4e508ca4.
