# Supplementary material for: When Average Isn't Good Enough: Identifying Meaningful Subgroups in Clinical Data
Source: Cognit Ther Res. 2024 Jan 28;48(4):537–51. doi: 10.1007/s10608-023-10453-x (PMC11341641; doi:10.1007/s10608-023-10453-x)
Supplement: Supplementary file 1 — Supplementary file1 (DOCX 957 kb) [file 10608_2023_10453_MOESM1_ESM.docx]

## Supplementary material

### Supplement A

When choosing the weights for the cluster analysis, we had to visually inspect different options to choose the weights that result in meaningful division of the data. To visualize the differences, examples of different weights for α and ß can be found below. The selection we made for our analysis was α = 1 and ß = 3.


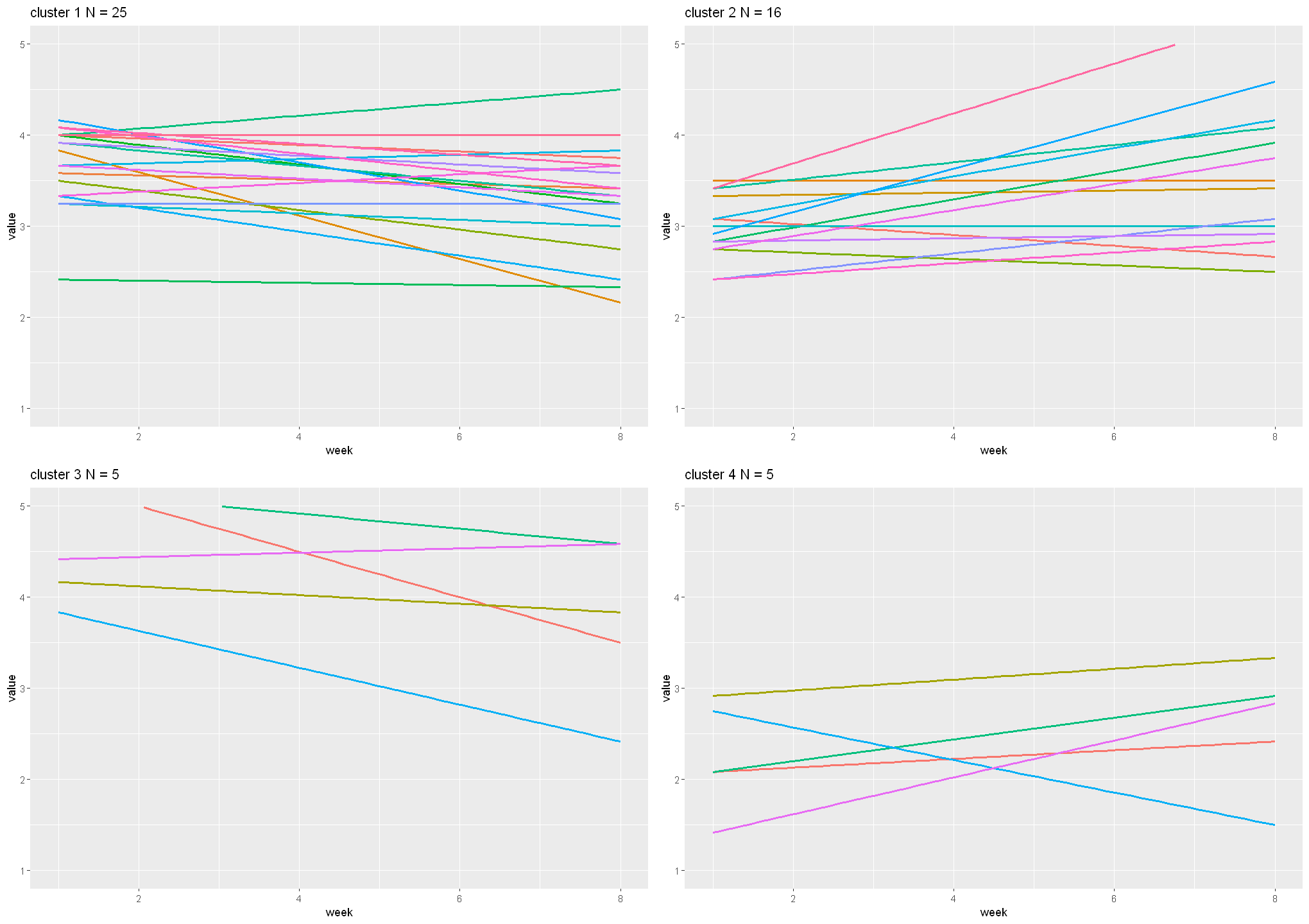


*Supplement A – Weights of α = 1 and ß = 1 for the clustering of item 1 (being present)*


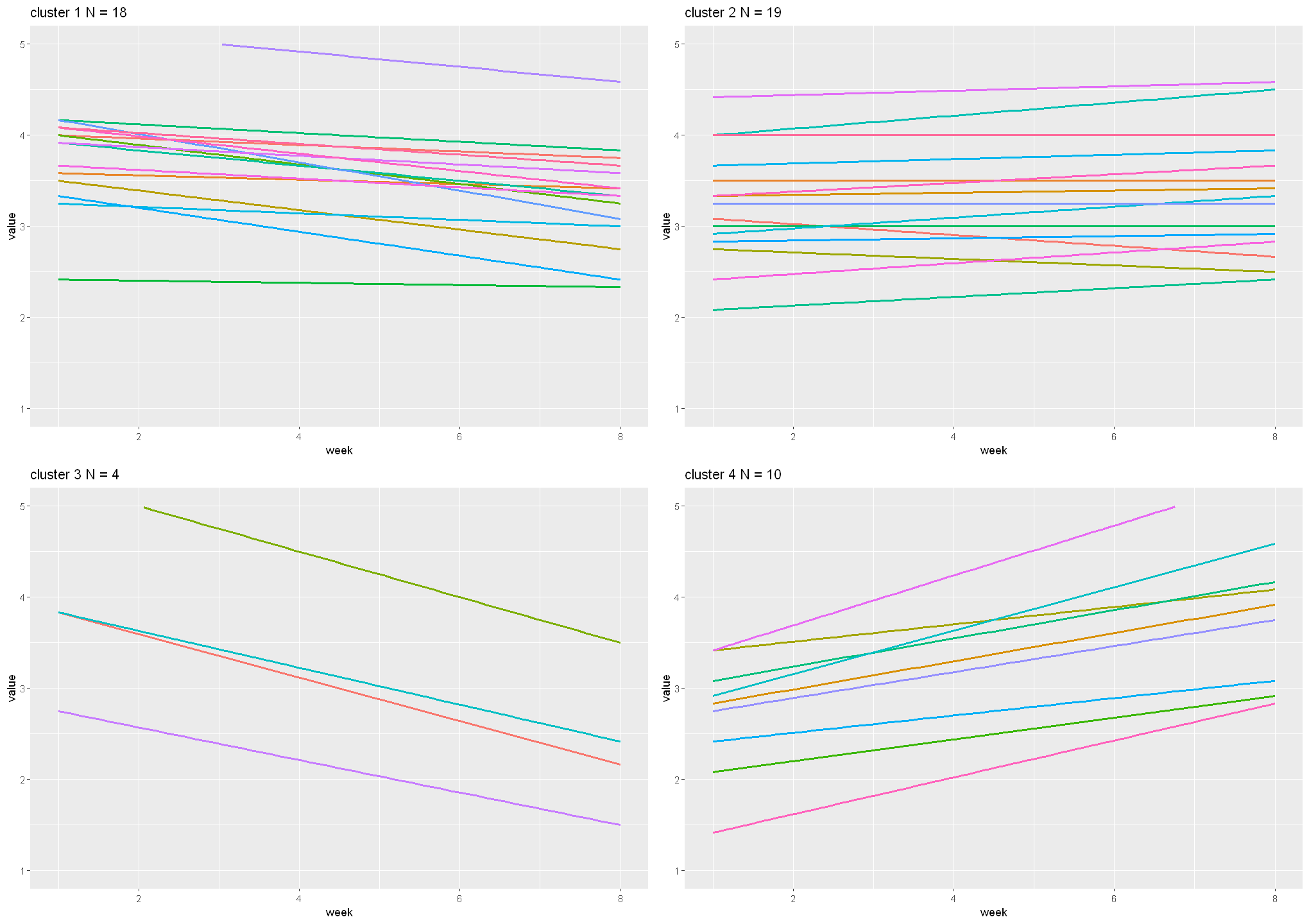


*Supplement A – Weights of α = 1 and ß = 3 for the clustering of item 1 (being present)*


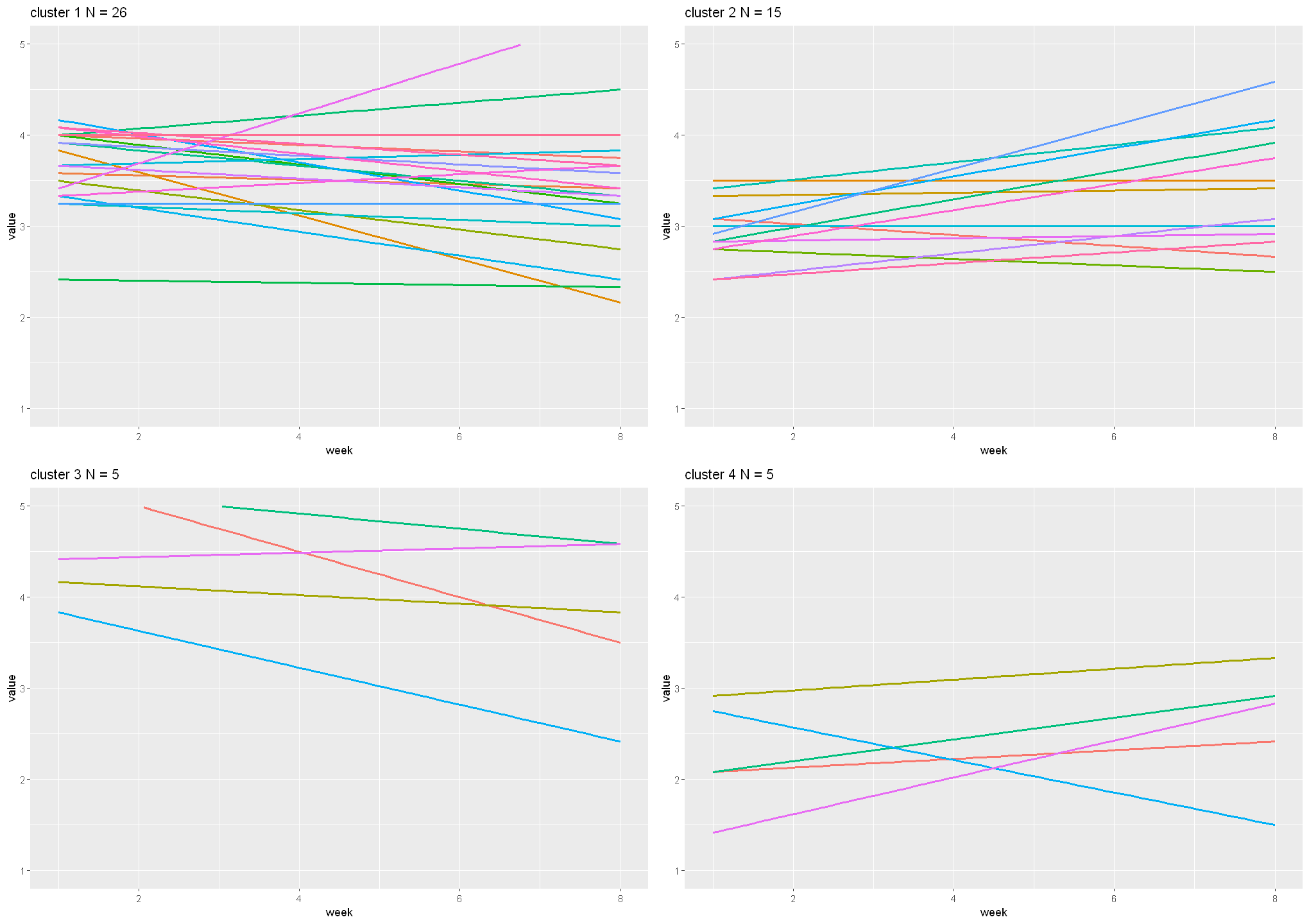


*Supplement A – Weights of α = 3 and ß = 1 for the clustering of item 1 (being present)*

### Supplement B

For the paper we showed the individual and aggregated regression lines for item 1 (being present) and item 7 (symptoms). However, the same conclusion can be reached from the analysis of the other items as well. The graphs for items 2 to 6 can be found below.


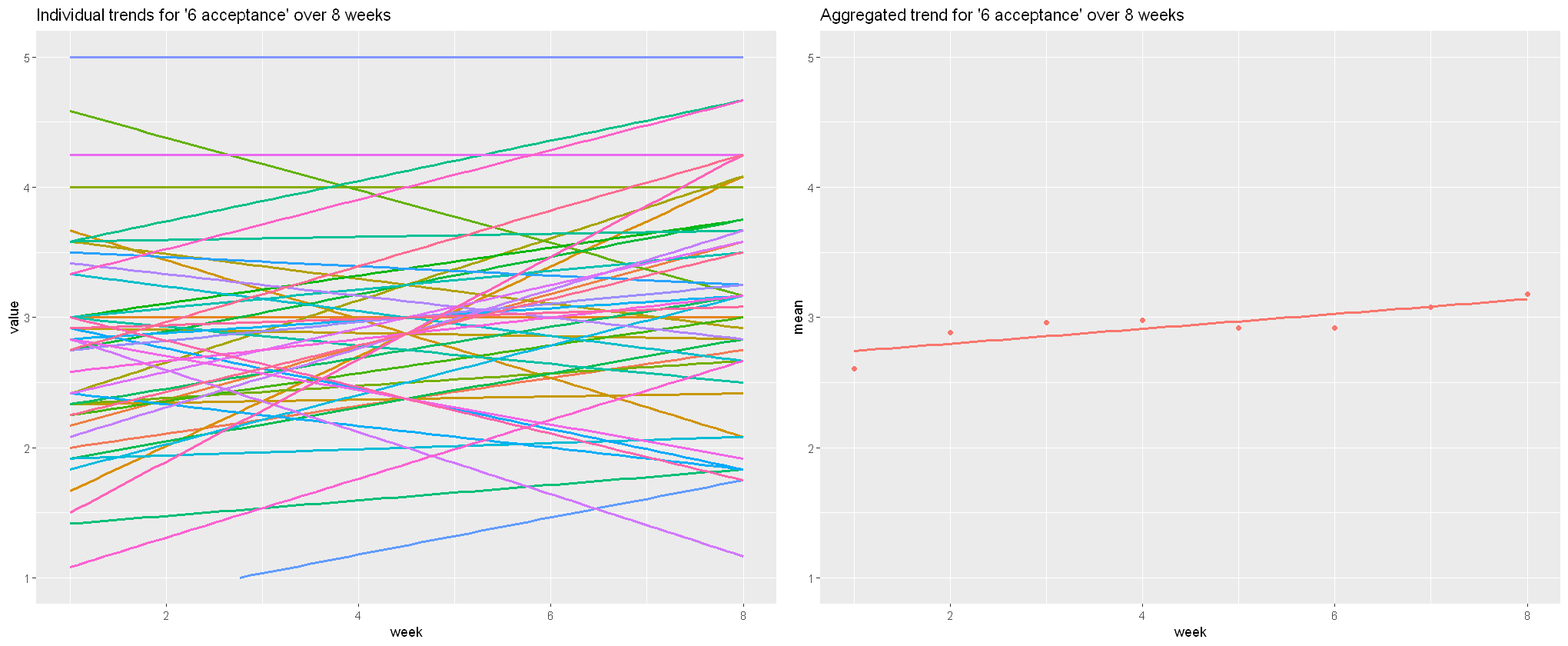


*Supplement B - Individual regression lines vs. aggregated regression line for the item 2 (acceptance)*


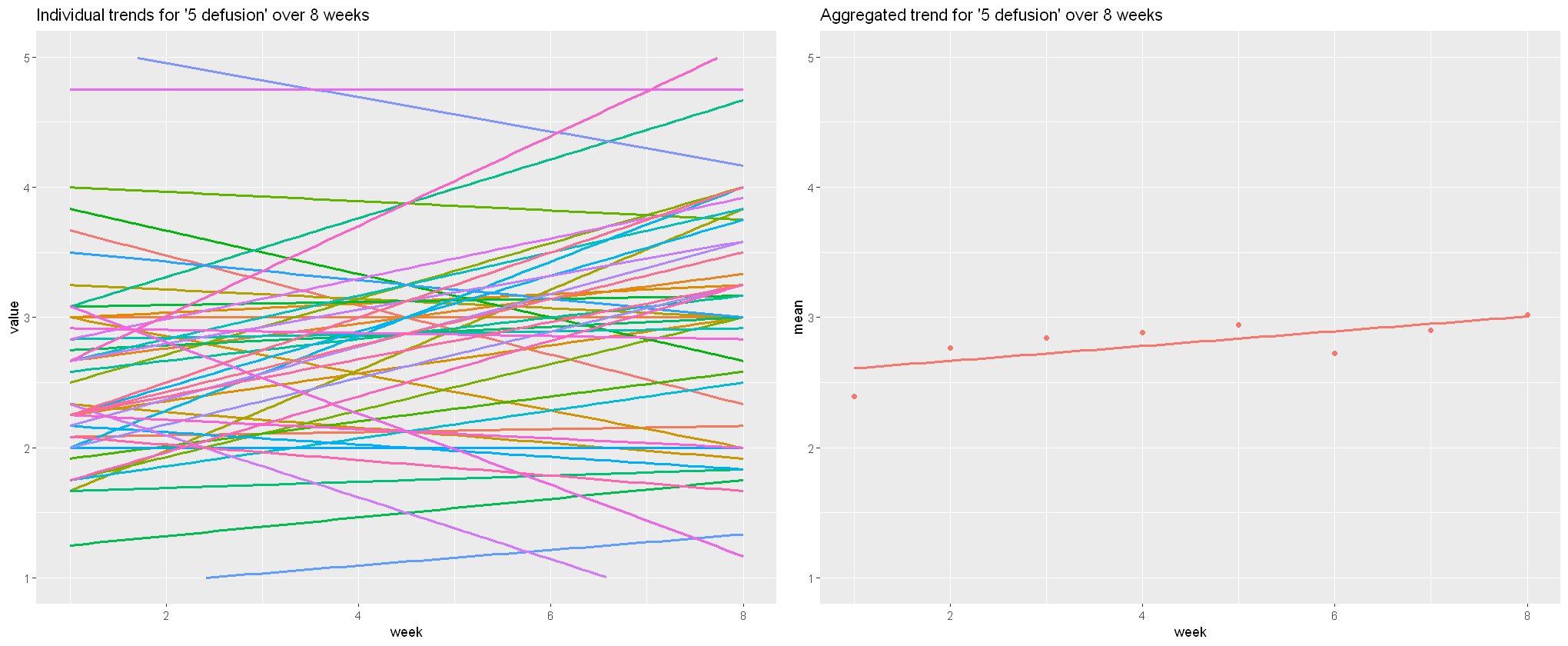


*Supplement B - Individual regression lines vs. aggregated regression line for the item 3 (defusion)*


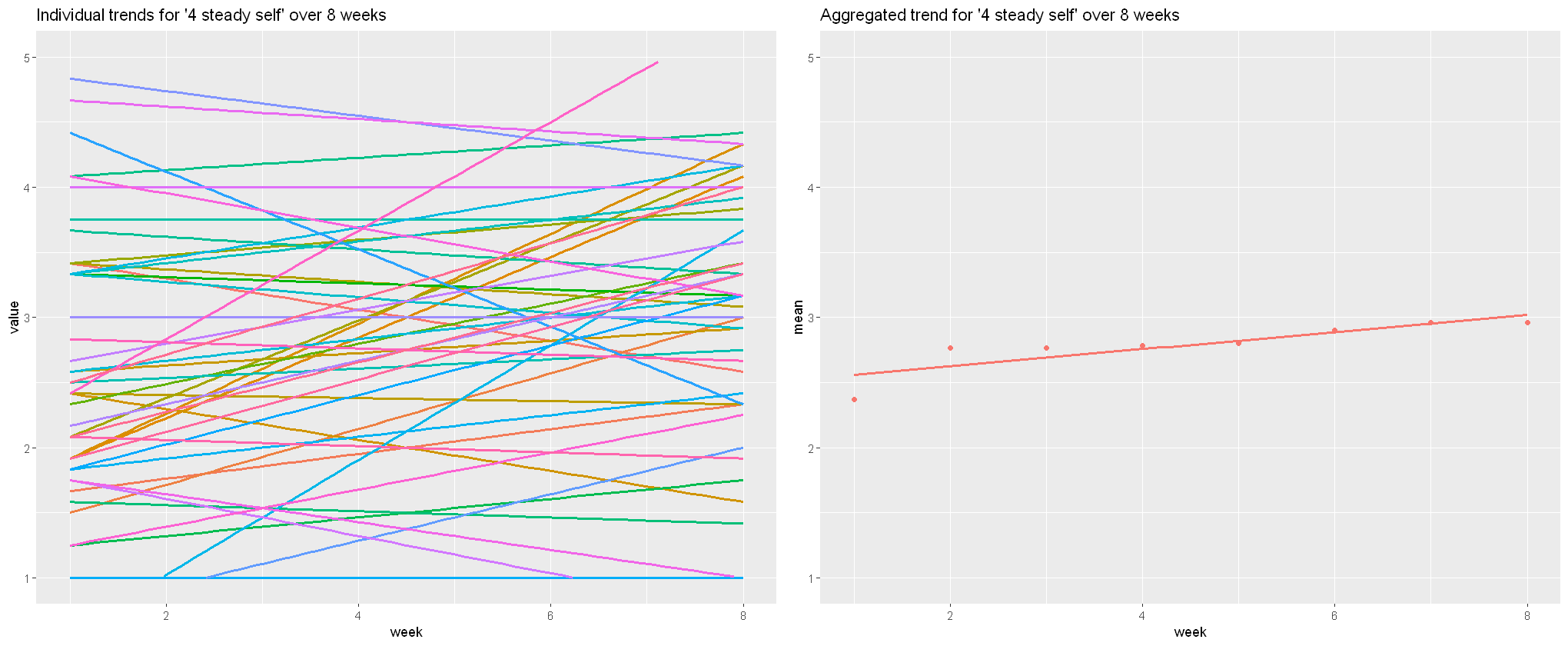


*Supplement B - Individual regression lines vs. aggregated regression line for the item 4 (steady self)*


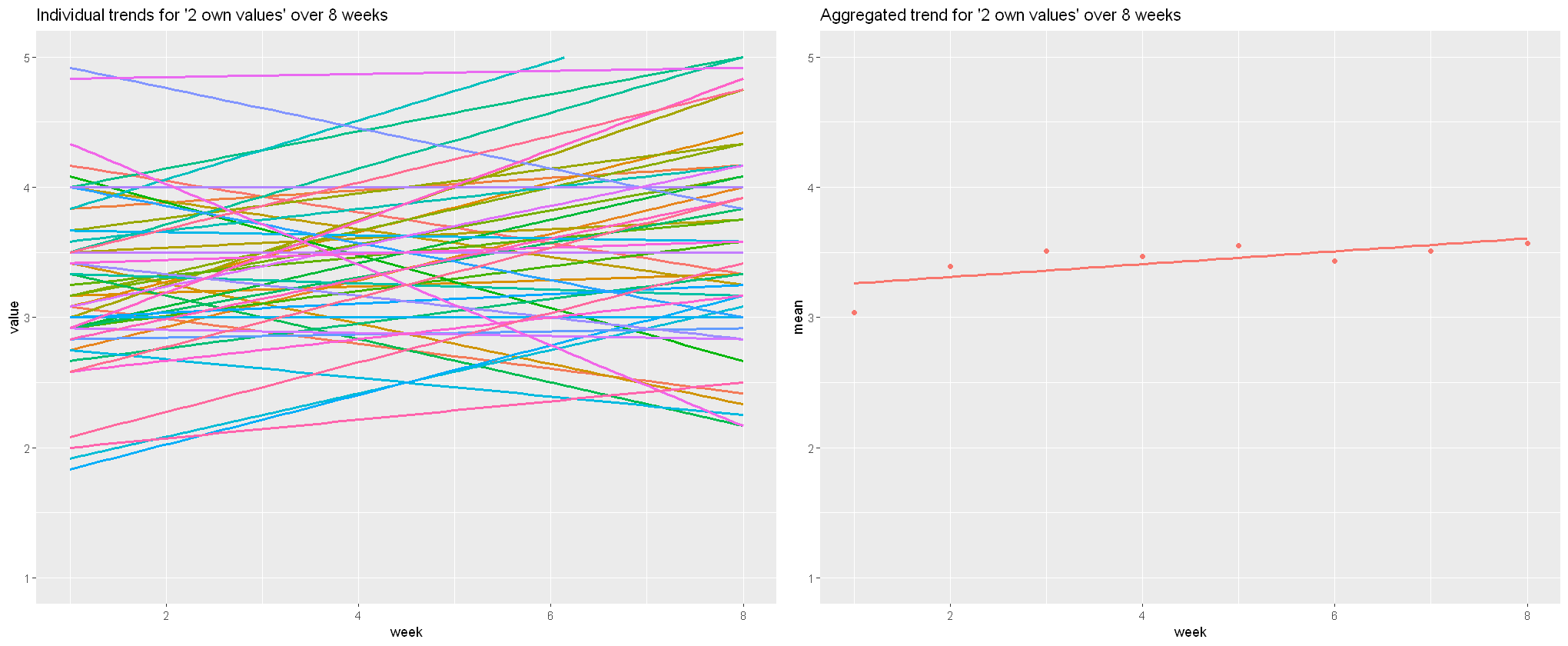


*Supplement B - Individual regression lines vs. aggregated regression line for the item 5 (own values)*


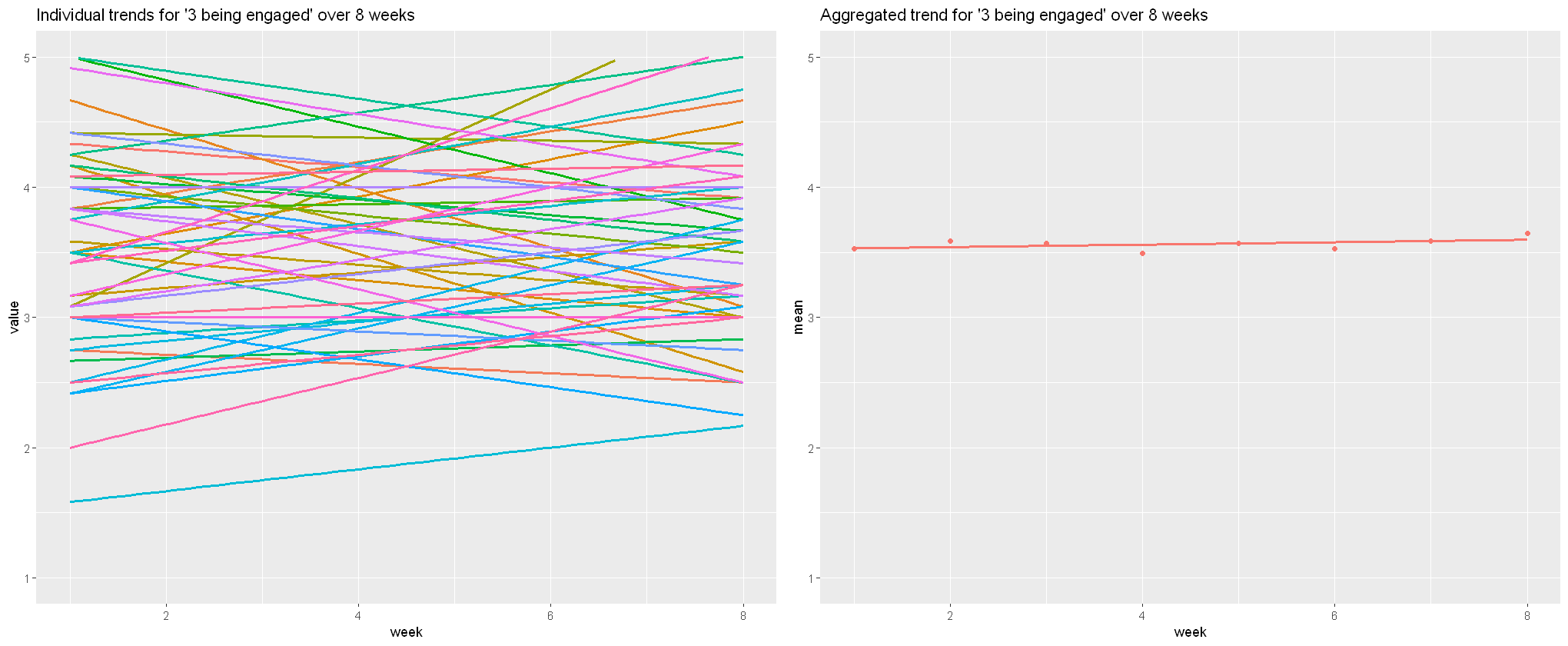


*Supplement B - Individual regression lines vs. aggregated regression line for the item 6 (being engaged)*

### Supplement C

The dendrogram below shows a typical top-down division using the Divisive Analysis Clustering (DIANA) technique. On the face of the dendrogram, the method seems to arrive at similar groups as Agglomerative Nesting Clustering (AGNES), but yields a lower coefficient, which is an indicator of the homogeneity of clusters. See supplement B for an example of the higher heterogeneity of DIANA-driven clusters.


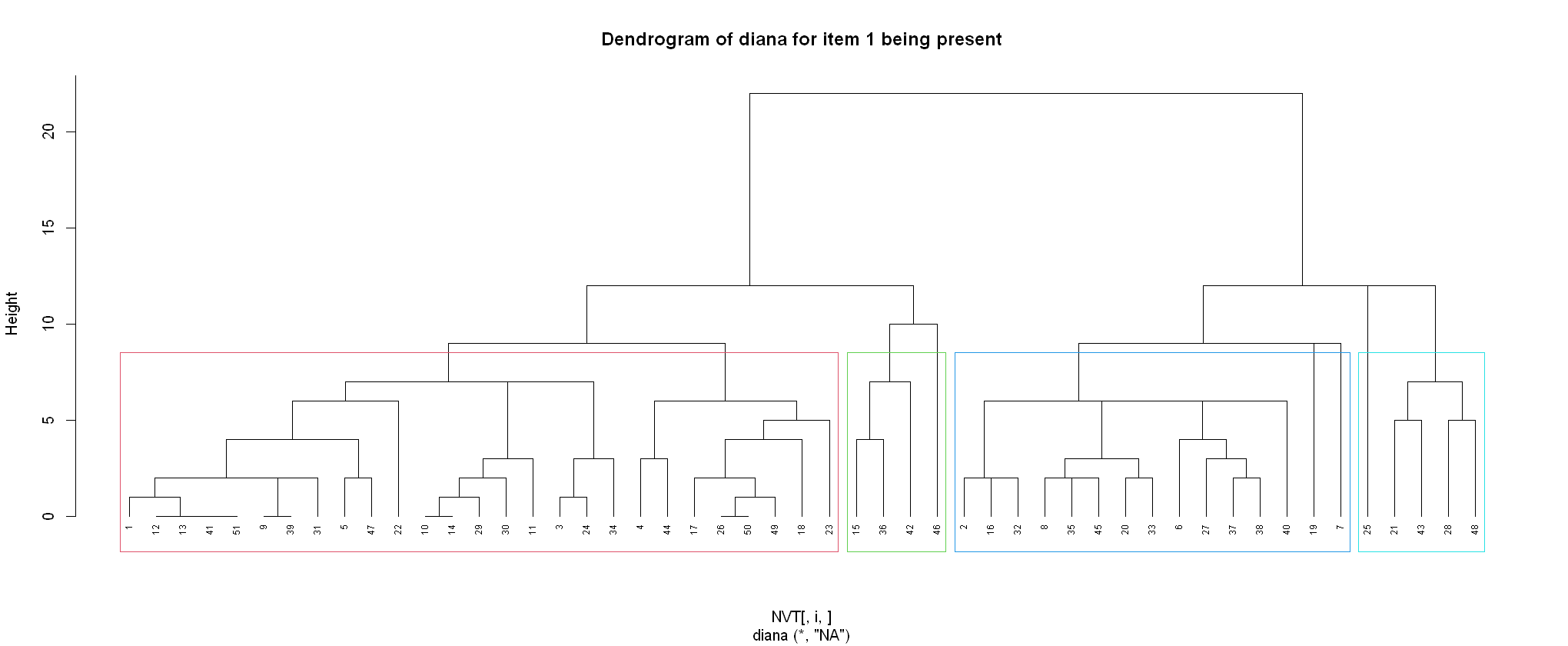


*Supplement C – DIANA Dendrogram for Item 1 (being present)*

**Supplement D**

The graph visualizes the linear regression lines per person per item across time separated by the four clusters calculated using Divisive Analysis Clustering (DIANA). The DIANA method arrived at heterogenous groups than does Agglomerative Nesting Clustering (AGNES) and was therefore regarded as the inferior clustering method for our purposes, which was to arrive at homogenous groups putting individuals with similar slopes and intercepts into groups.


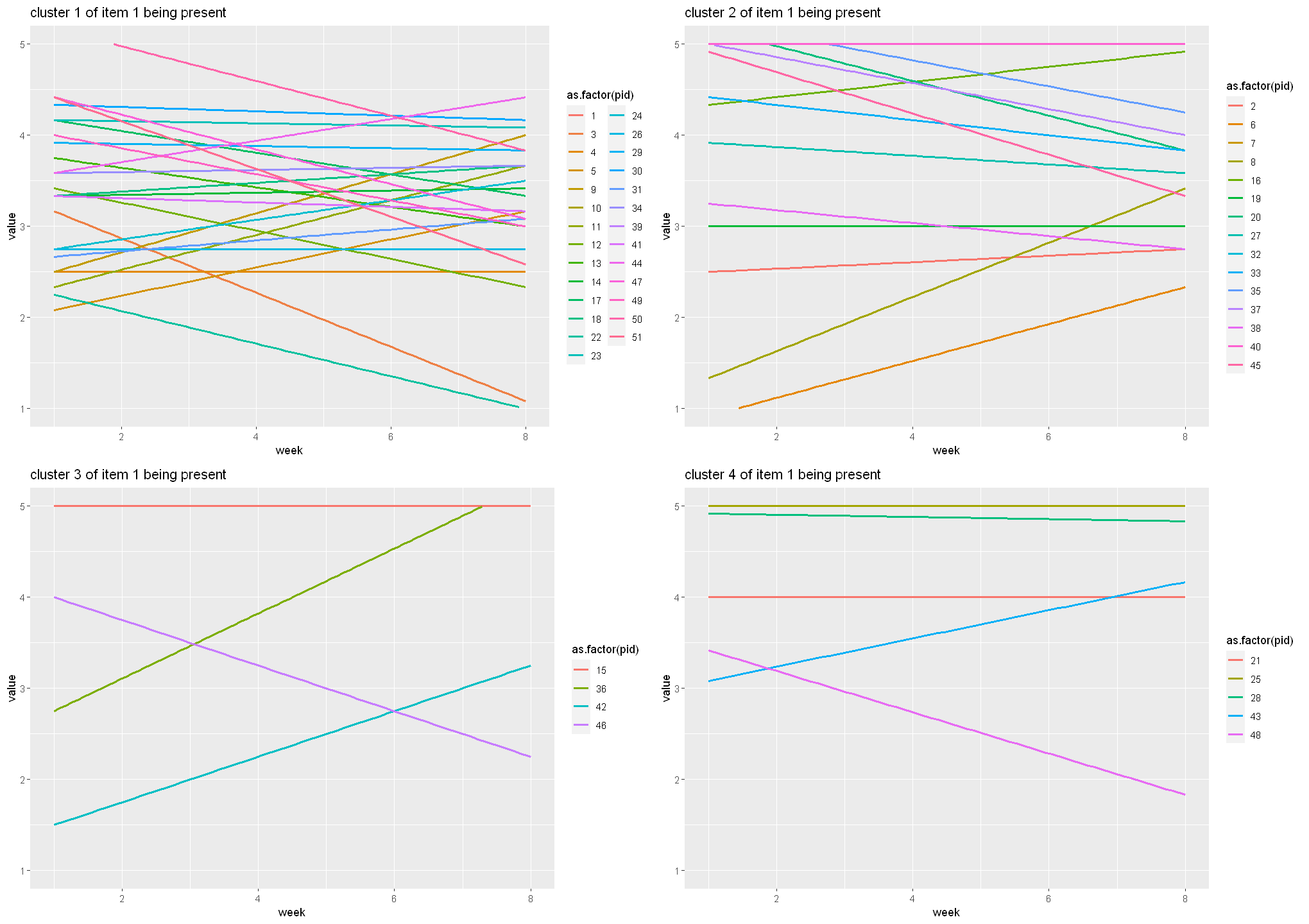


*Supplement D -* *DIANA Clusters for Item 1 (being present)*

### Supplement E

For the purpose of this paper, we have opted for using Item 1 (being present) as the example, but we did calculate AGNES dendrograms and clusters for all available items. It is important to note that the proposed method of clustering similar trajectories together produces the most homogenous groups possible but changes the meaning of the result for each item. We use the example of groups by their trajectories across time in their subjective appraisal of being able to stay in the present moment (or be mindful). Below the AGNES dendrograms for Items 2-7 are presented that all could be used for the same argument, albeit with different content.


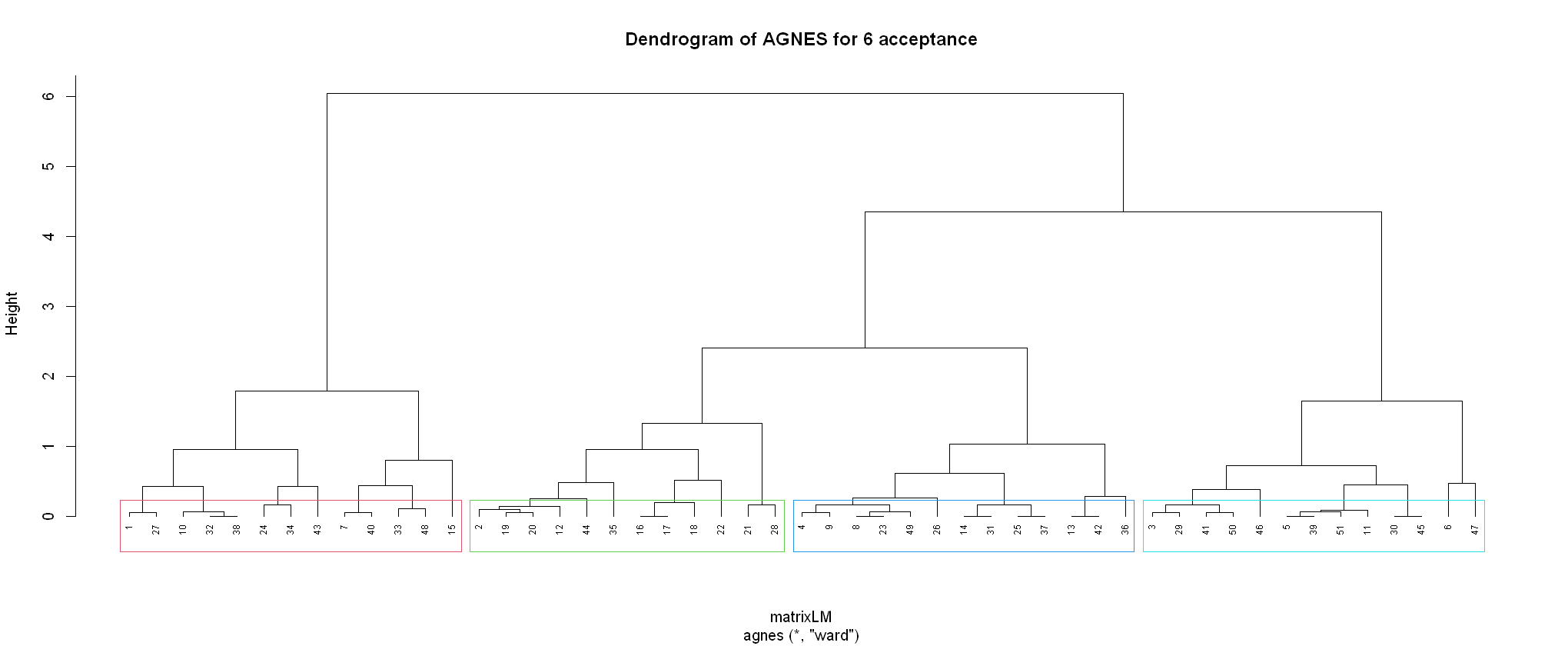


*Supplement E –* *AGNES Dendrogram for Item 2 (acceptance)*


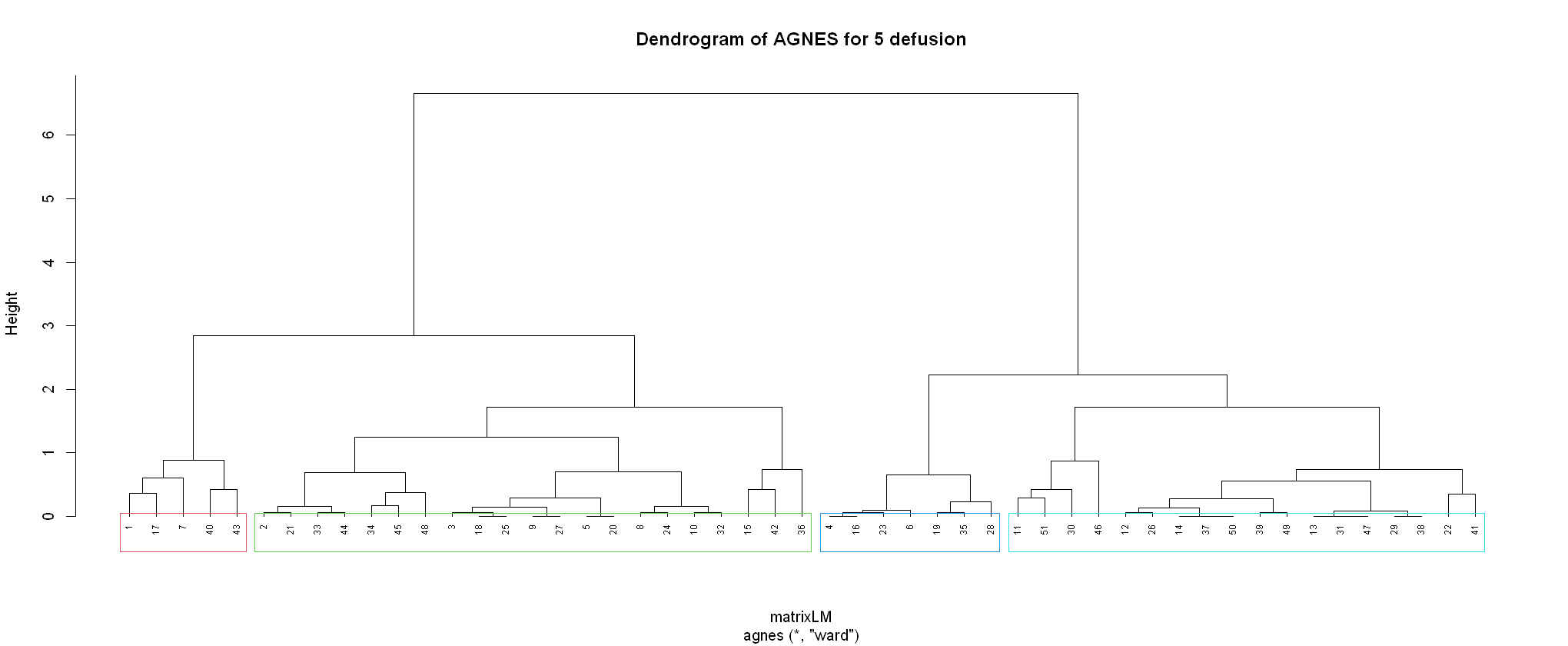


*Supplement E –* *AGNES Dendrogram for Item 3 (defusion)*


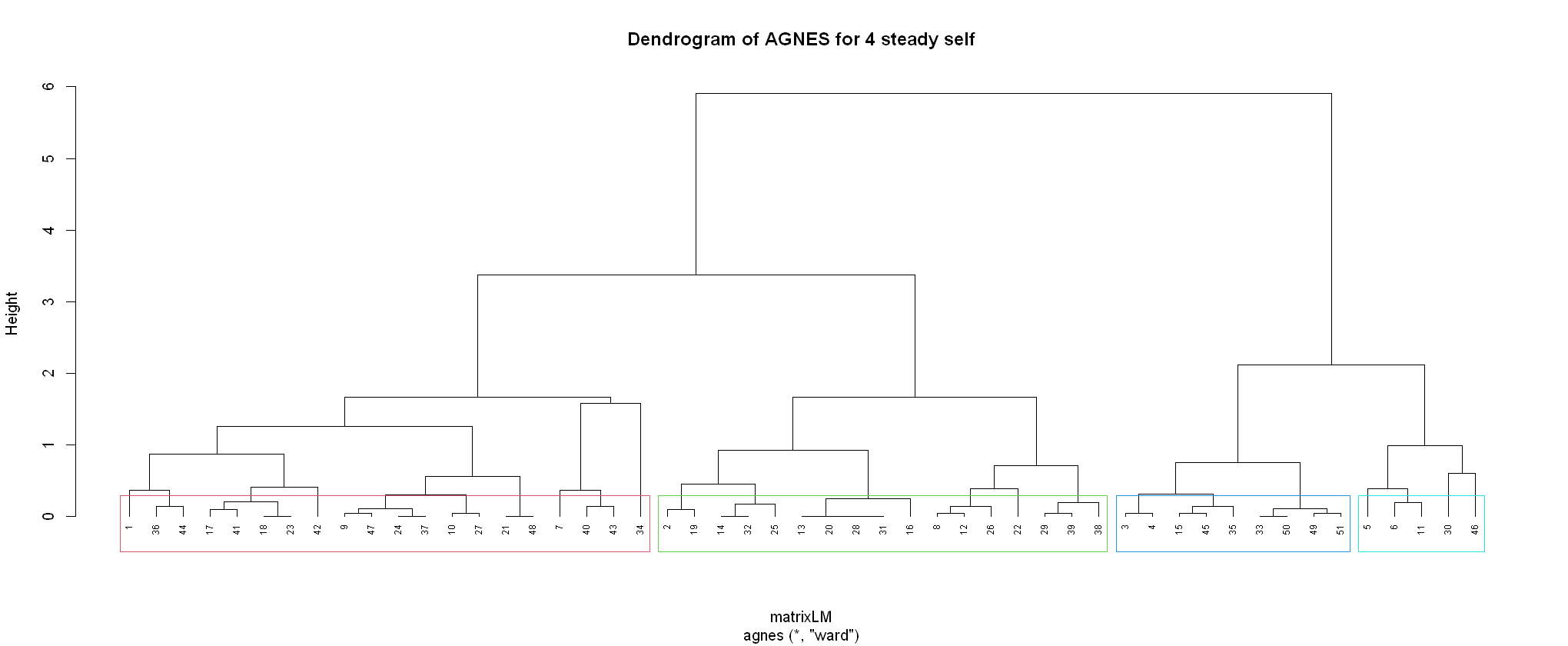


*Supplement E –* *AGNES Dendrogram for Item 4 (steady self)*


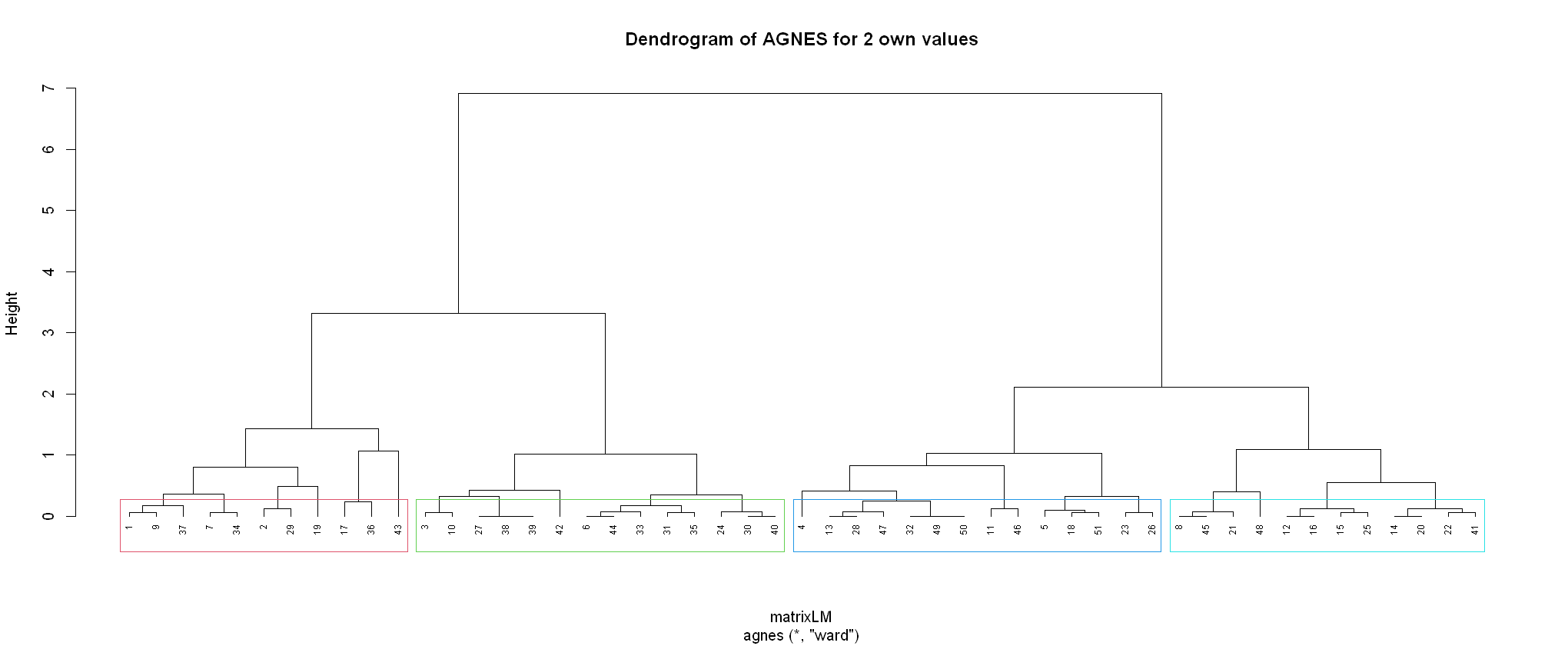


*Supplement E –* *AGNES Dendrogram for Item 5 (own values)*


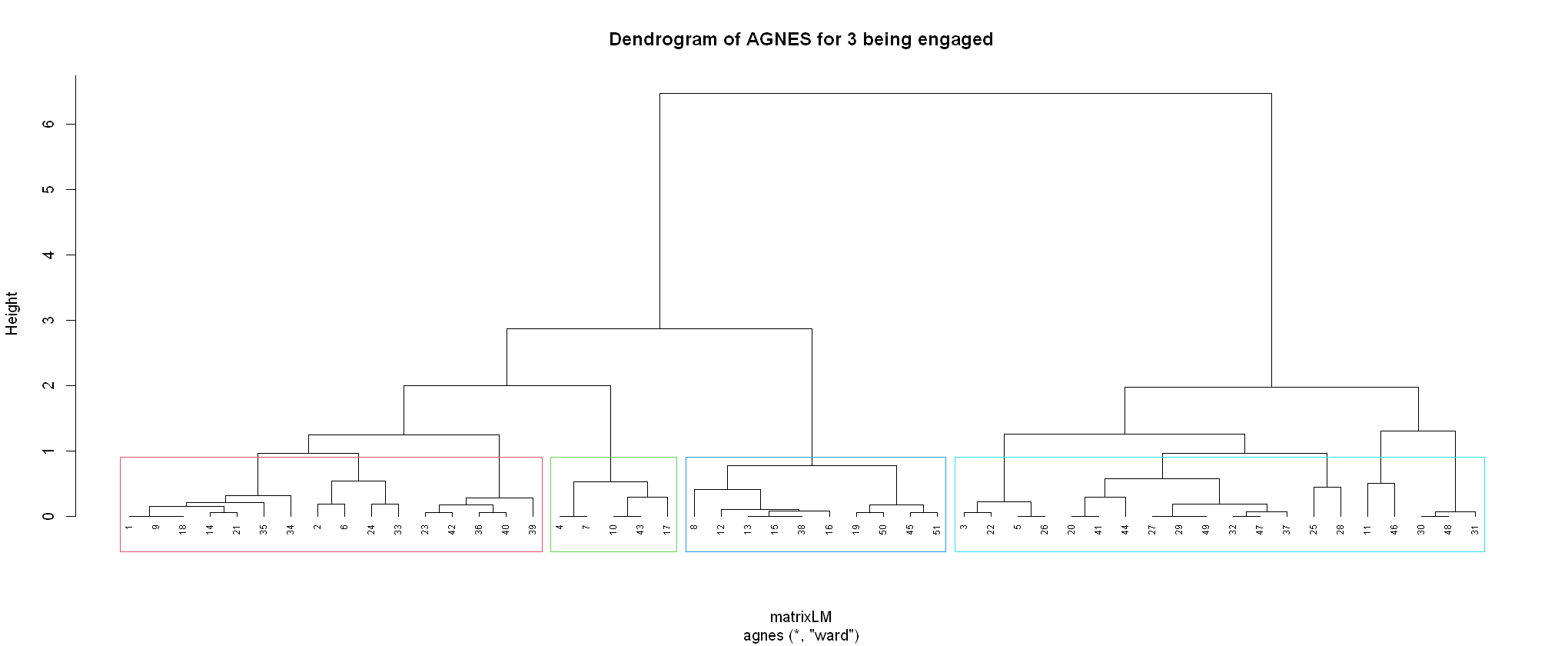


*Supplement E –* *AGNES Dendrogram for Item 6 (being enganged)*


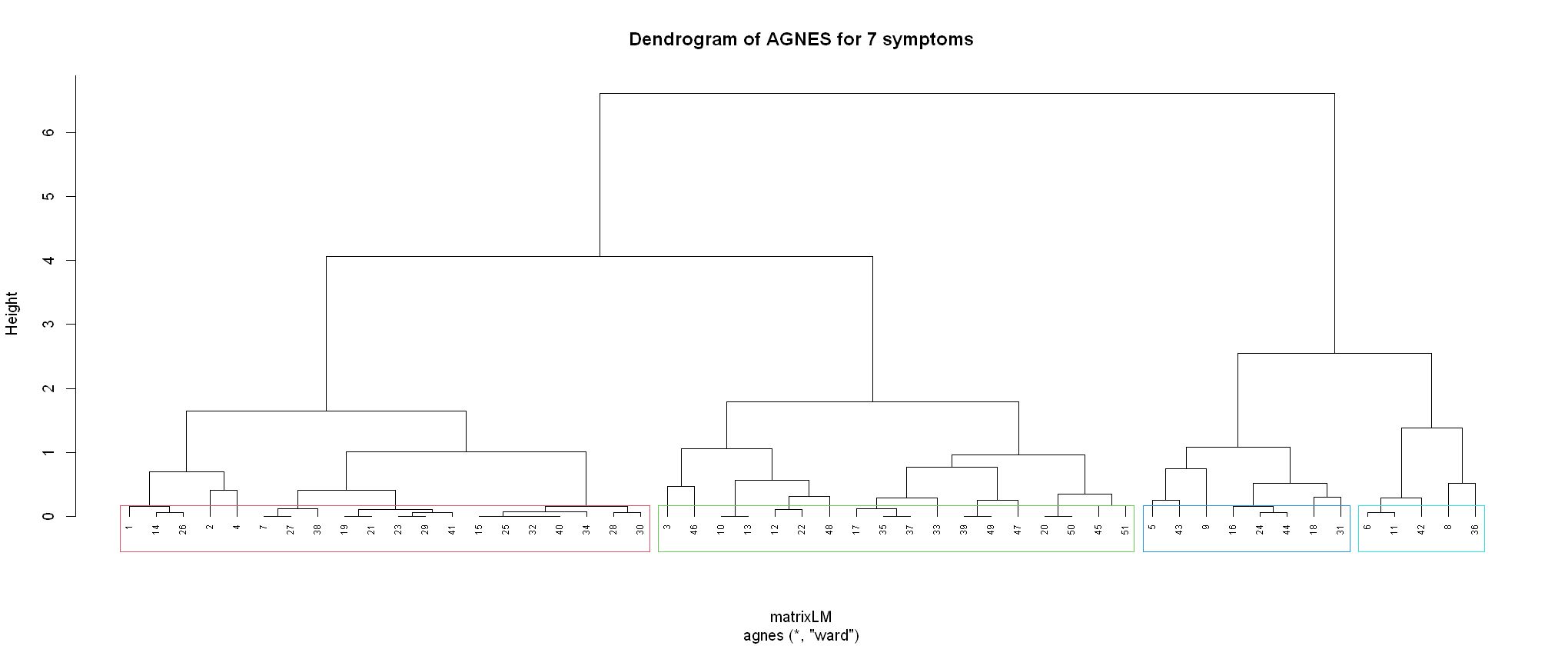


*Supplement E –* *AGNES Dendrogram for Item 7 (symptoms)*

### Supplement F

For the purpose of this paper, we have opted for using Item 1 (being present) as the example but we calculated the results for each item. Hence, the linear regression lines per person per item across time separated by the four AGNES clusters are presented below to visualize the results of the AGNES clustering used in the paper. The graphs show how the clustering algorithm arrives at sorting the individual linear regression lines into homogenous groups, putting more weight on the slope than on the intercept.


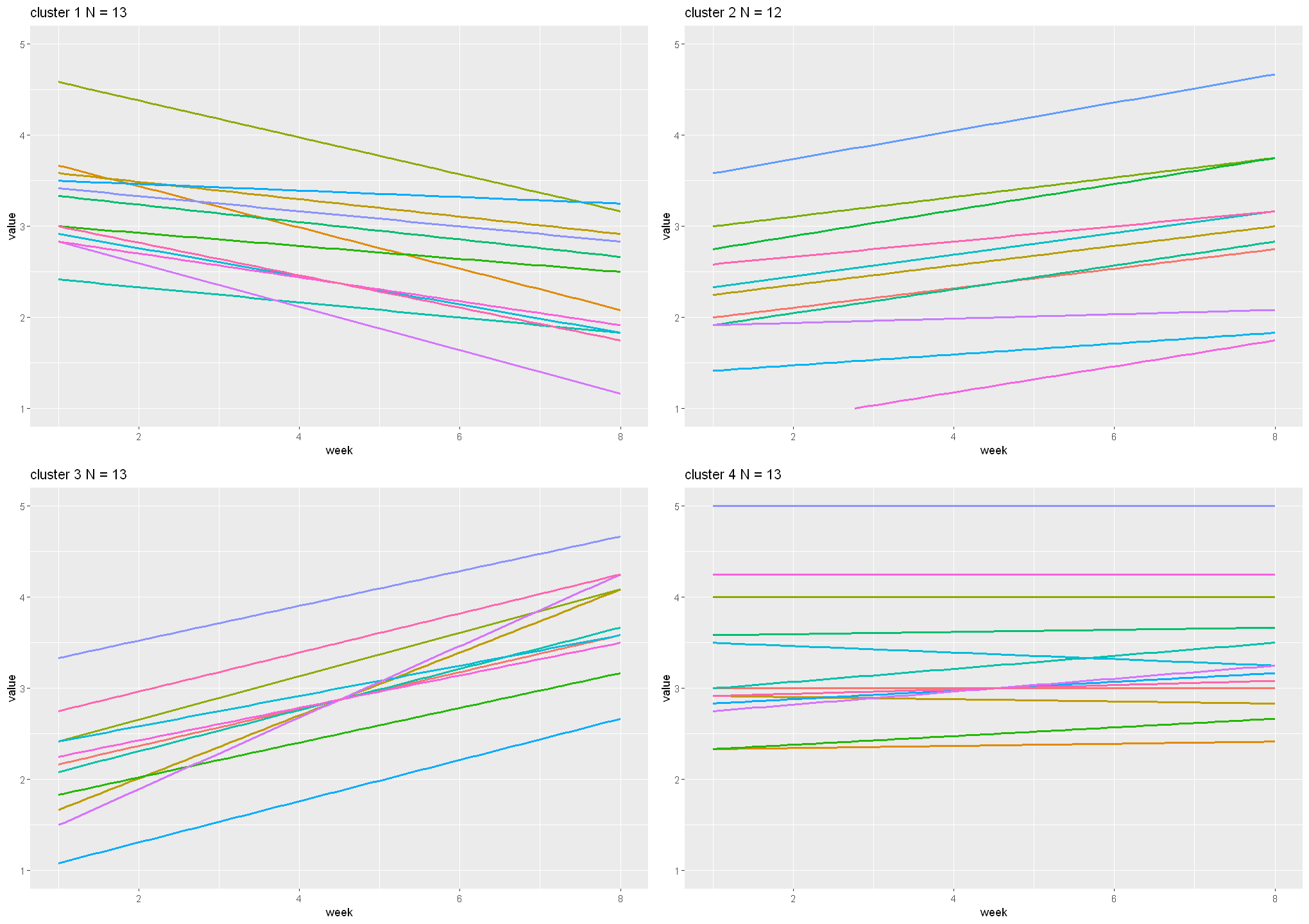


*Supplement F –* *AGNES Clusters for Item 2 (acceptance)*


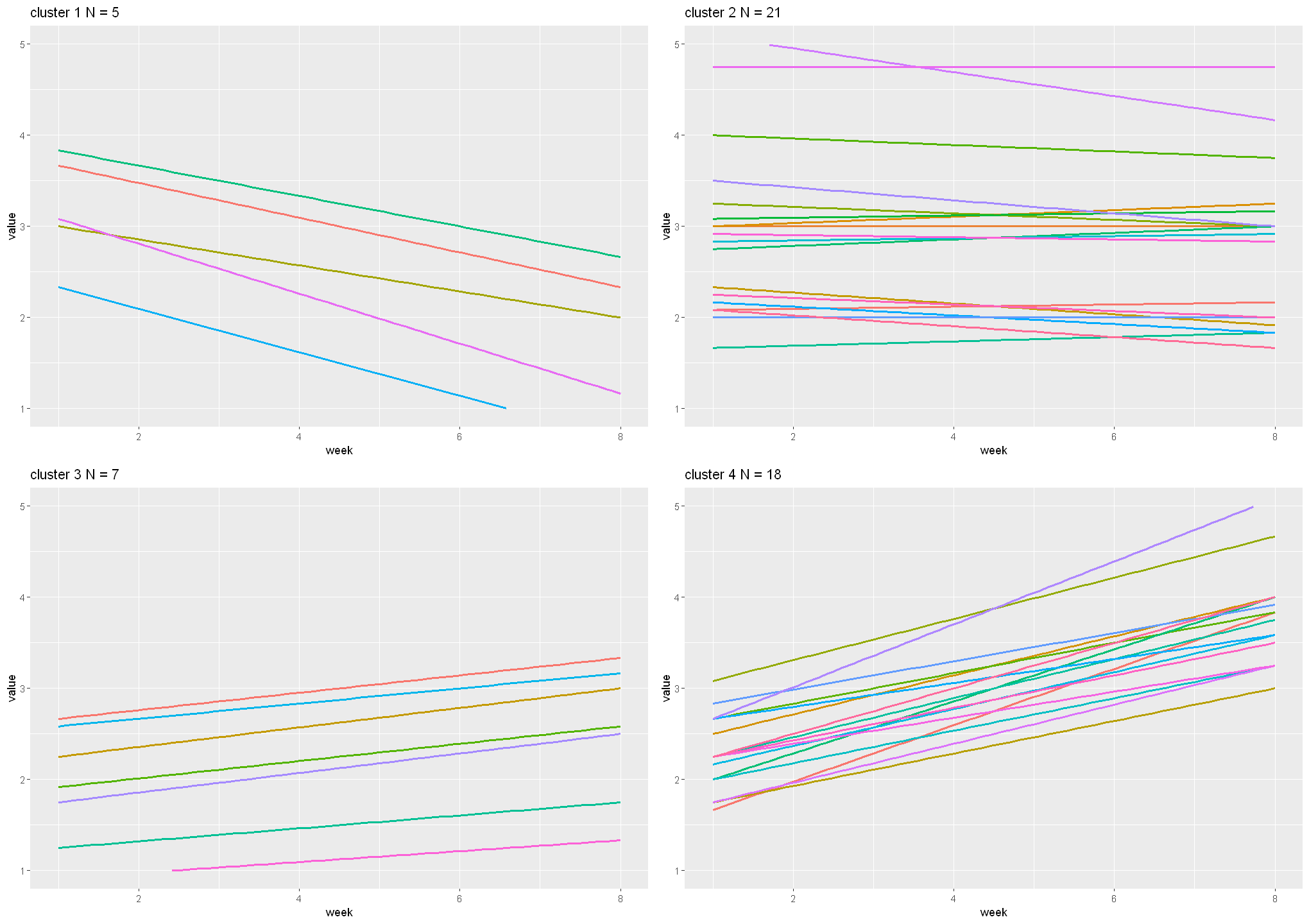


*Supplement F –* *AGNES Clusters for Item 3 (defusion)*


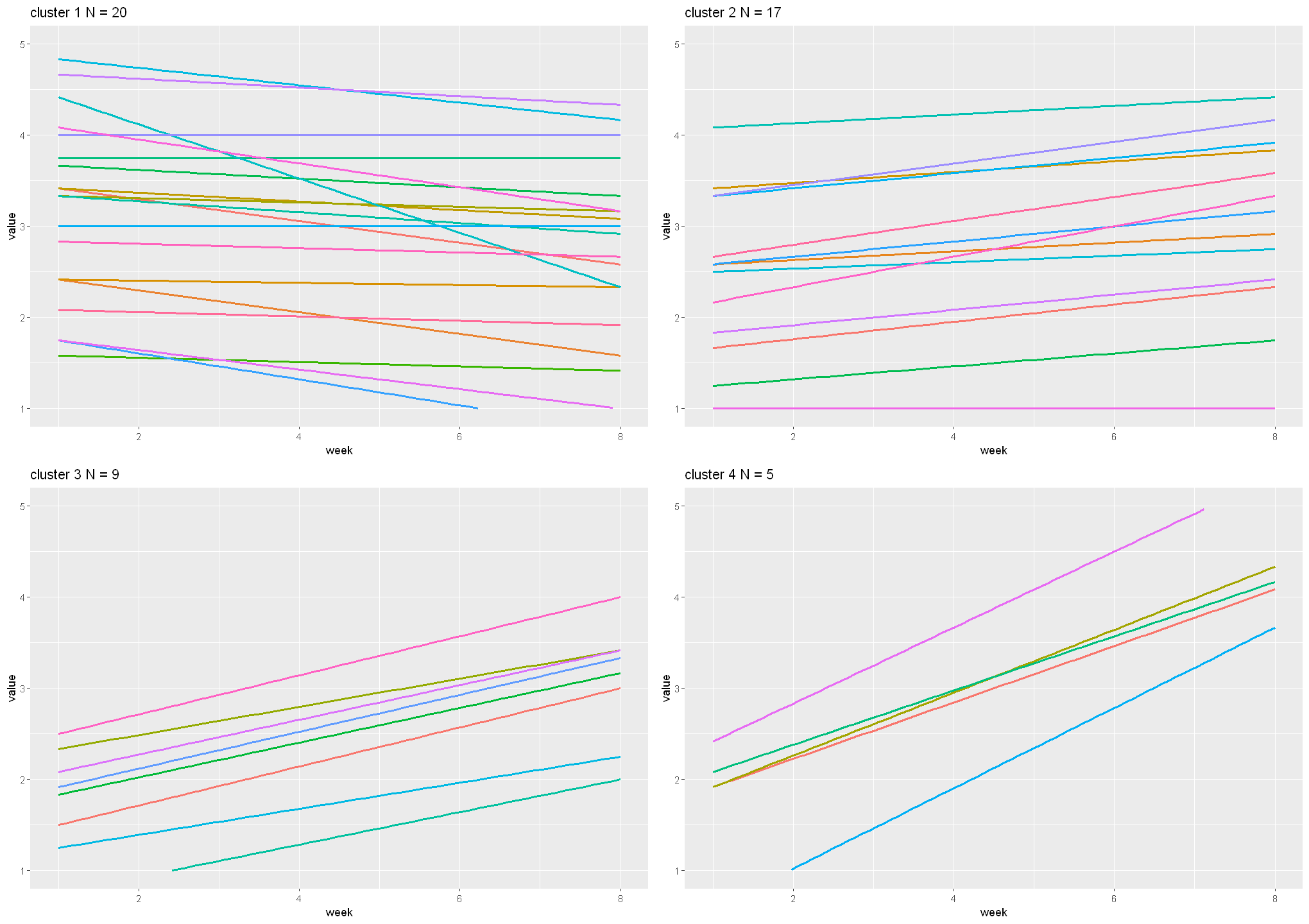


*Supplement F –* *AGNES Clusters for Item 4 (steady self)*


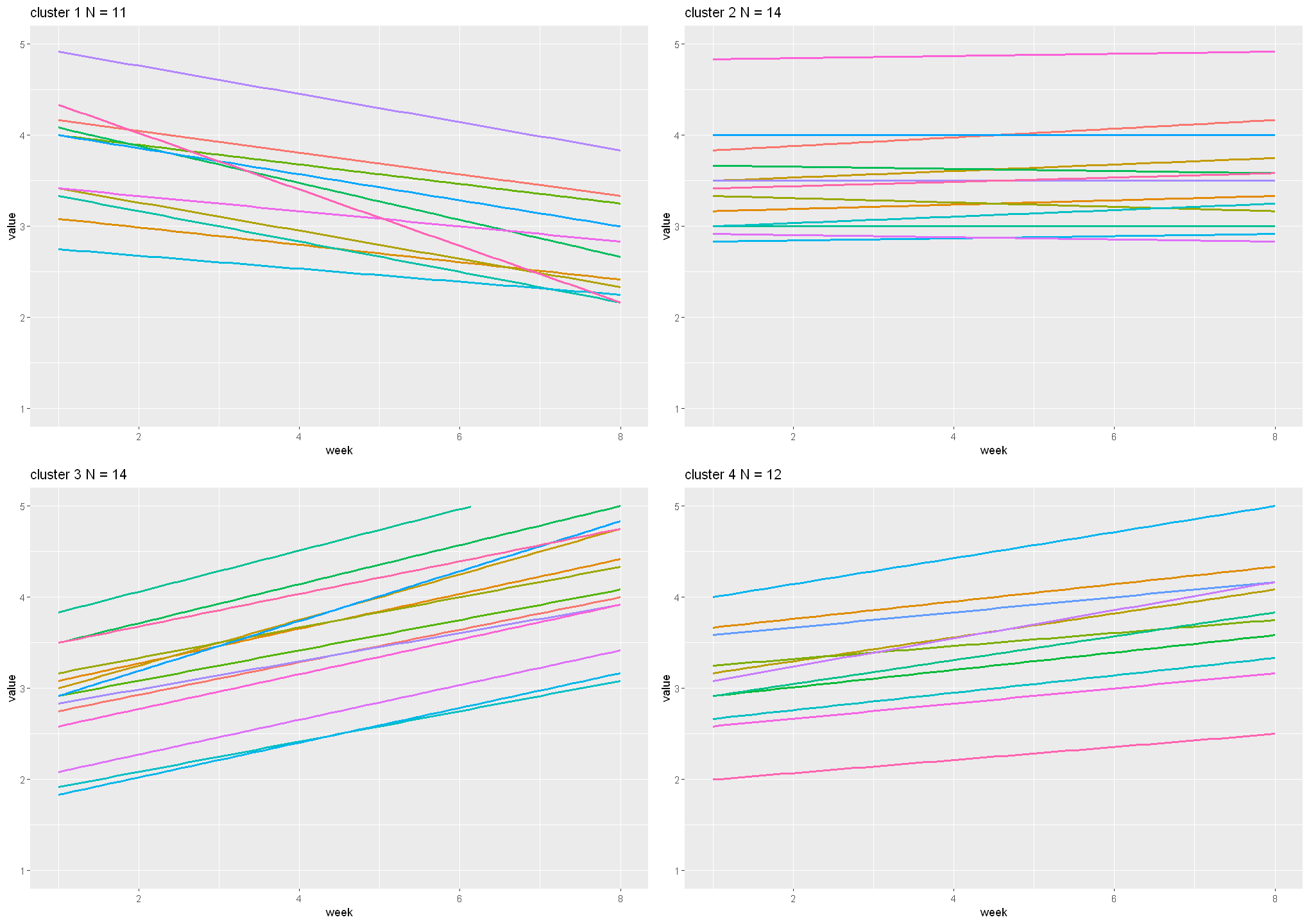


*Supplement F –* *AGNES Clusters for Item 5 (own values)*


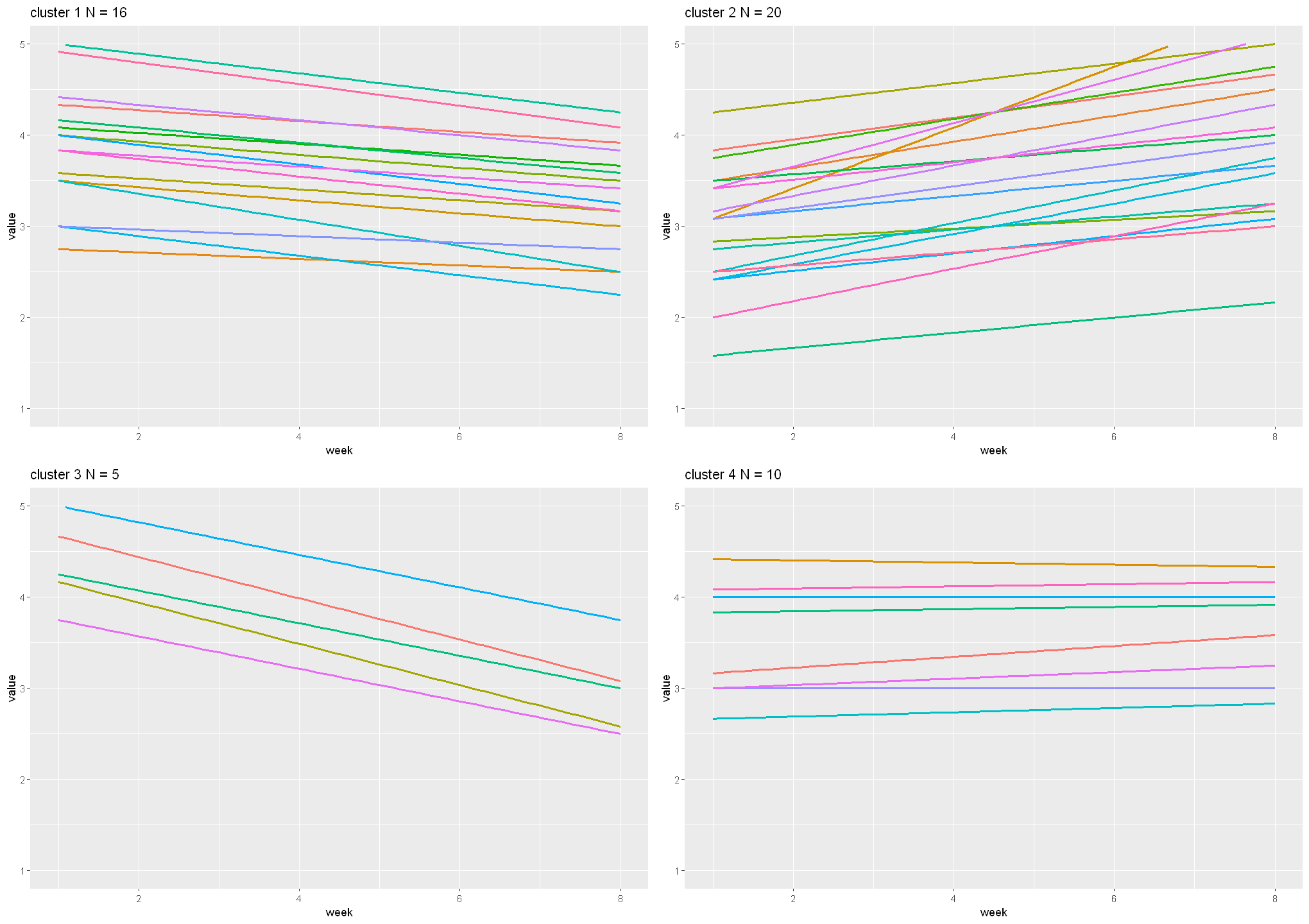


*Supplement F –* *AGNES Clusters for Item 6 (being engaged)*


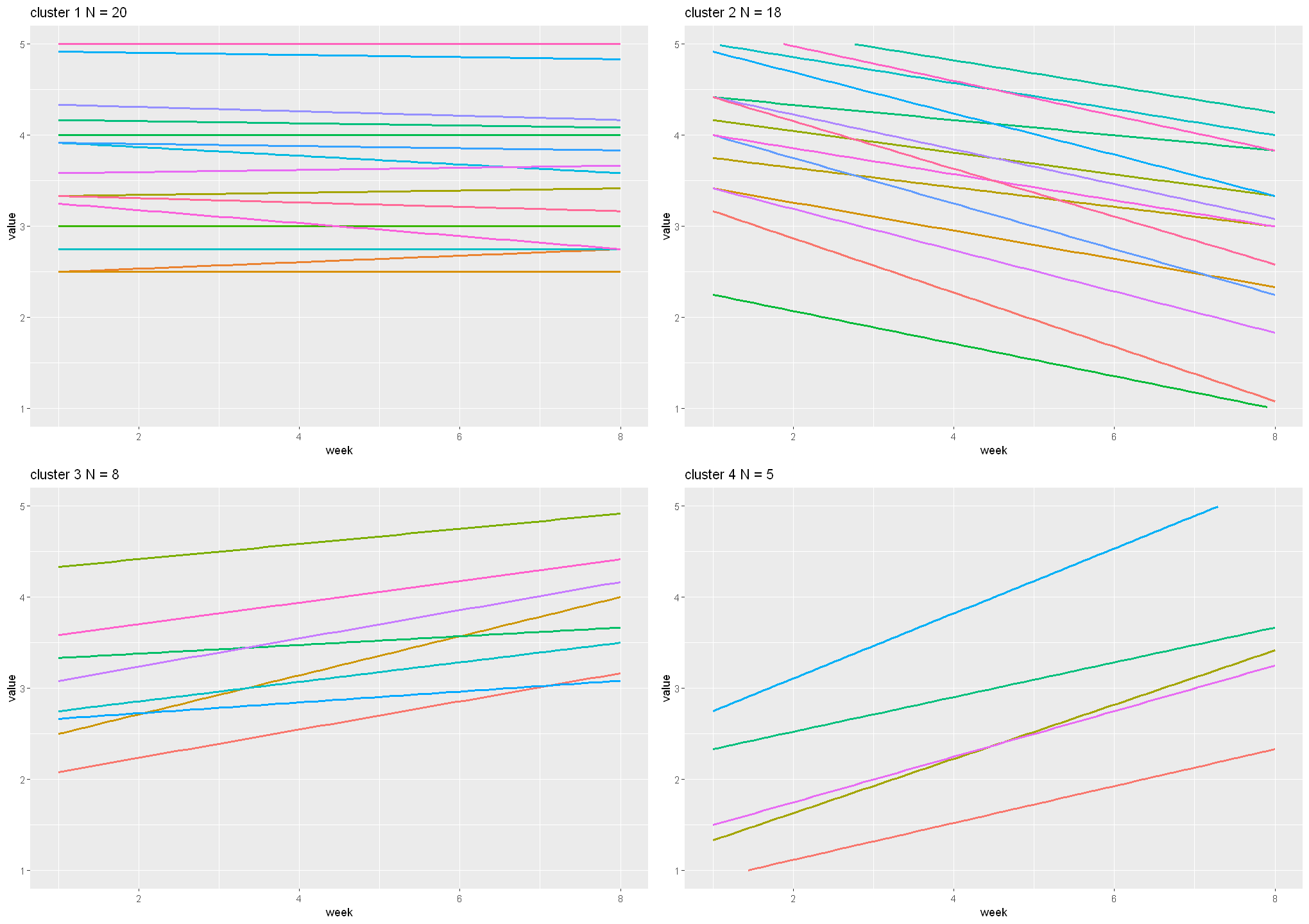


*Supplement F –* *AGNES Clusters for Item 7 (symptoms)*

### Supplement G

For the purpose of this paper, we have opted for using Item 1 (being present) as the example but we calculated the results for each item. Hence, the Cluster time series lines per item across time separated by the four AGNES clusters are presented below. The graphs show the changes across the weeks and which cluster is improving, worsening, or staying the same. Furthermore, sudden changes are also visible in this week-by-week visualization.


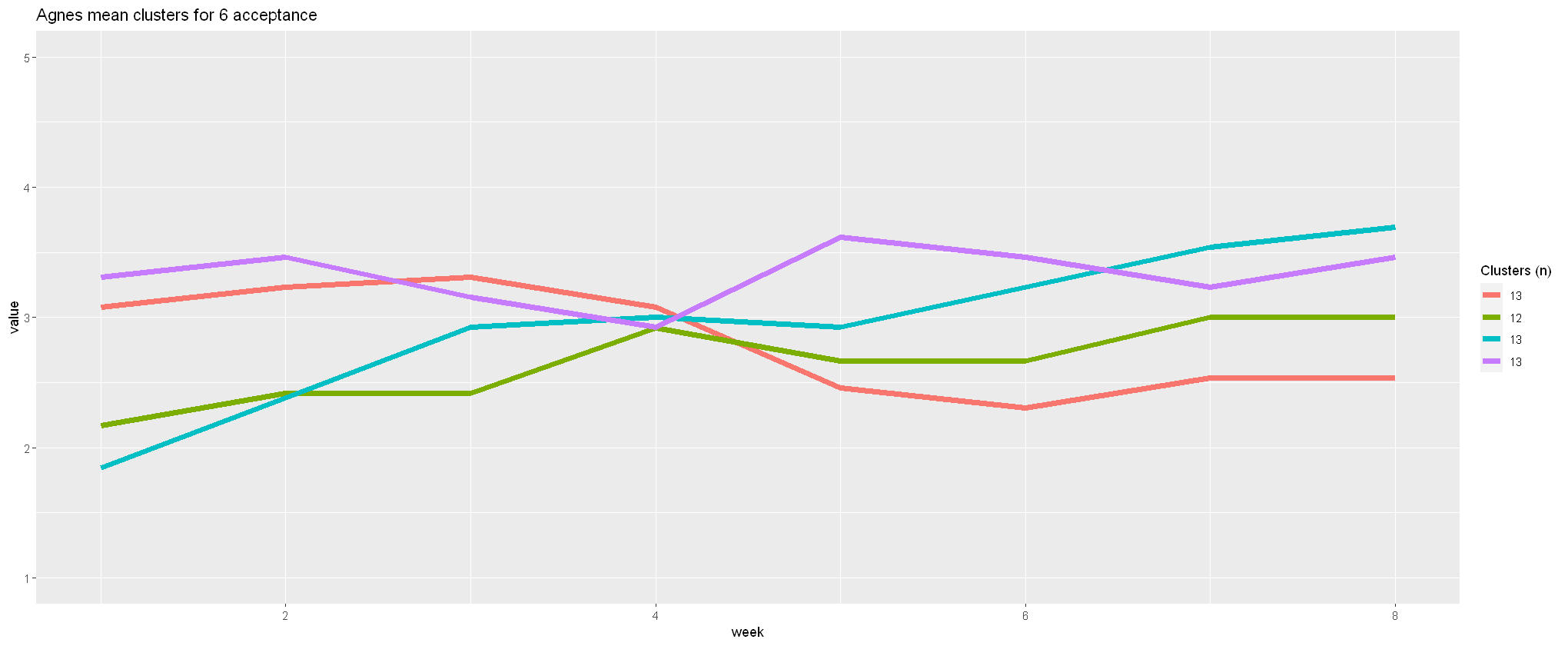
*Supplement G - Average Cluster Time Series for Item 2 (acceptance)*


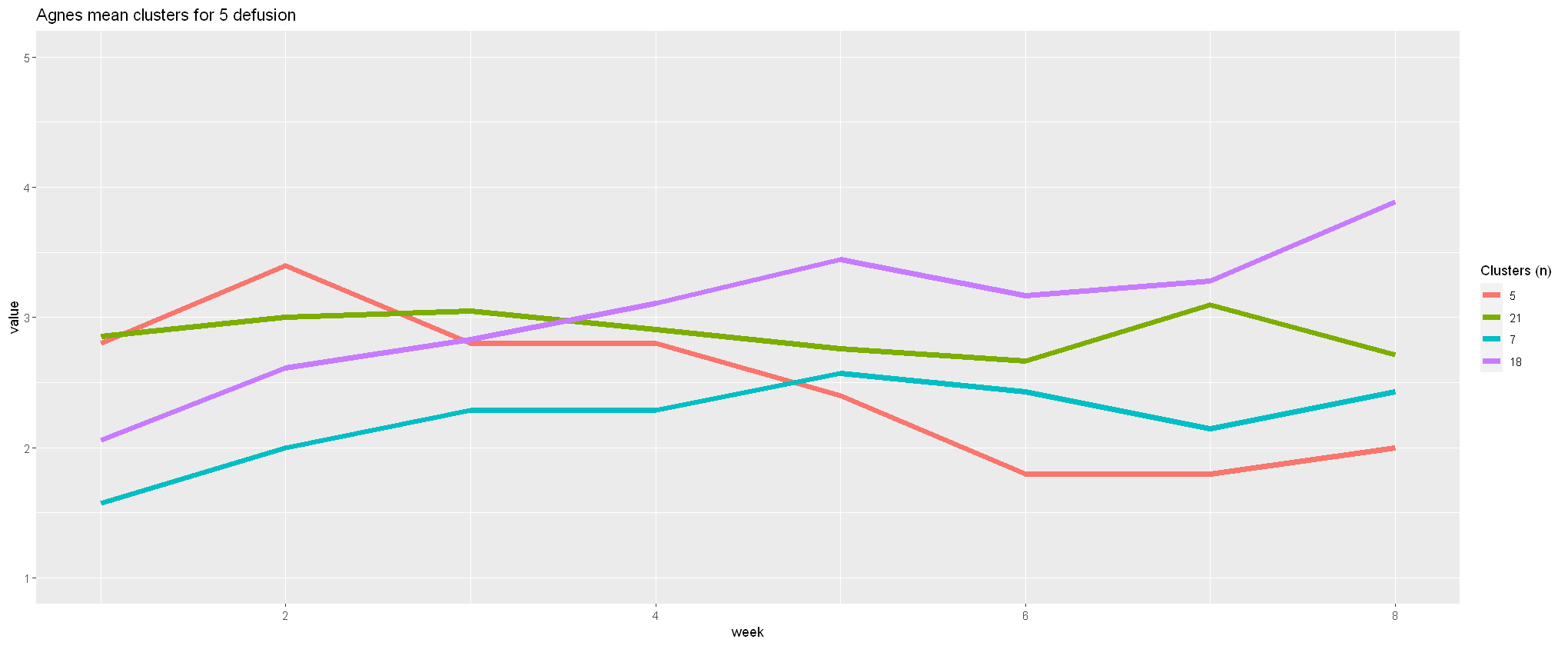


*Supplement G - Average Cluster Time Series for Item 3 (defusion)*


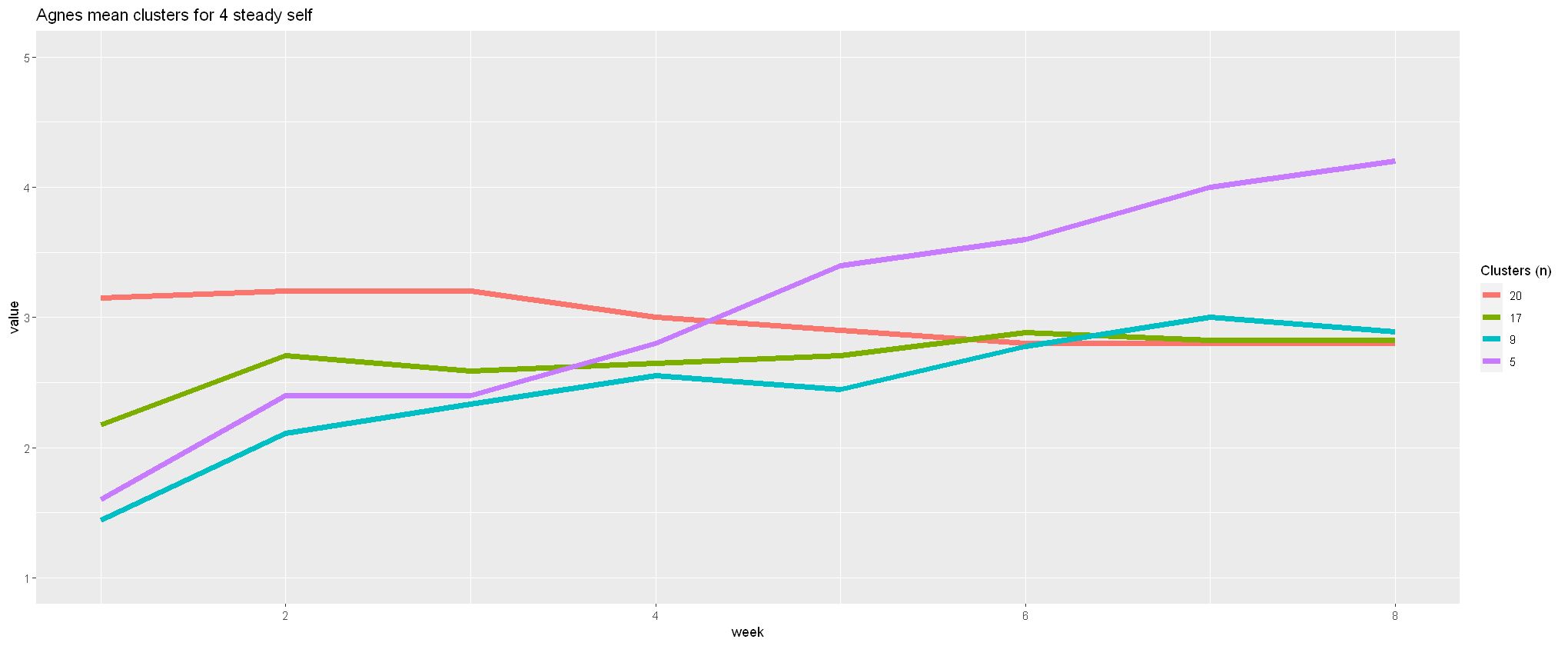


*Supplement G - Average Cluster Time Series for Item 4 (steady self)*


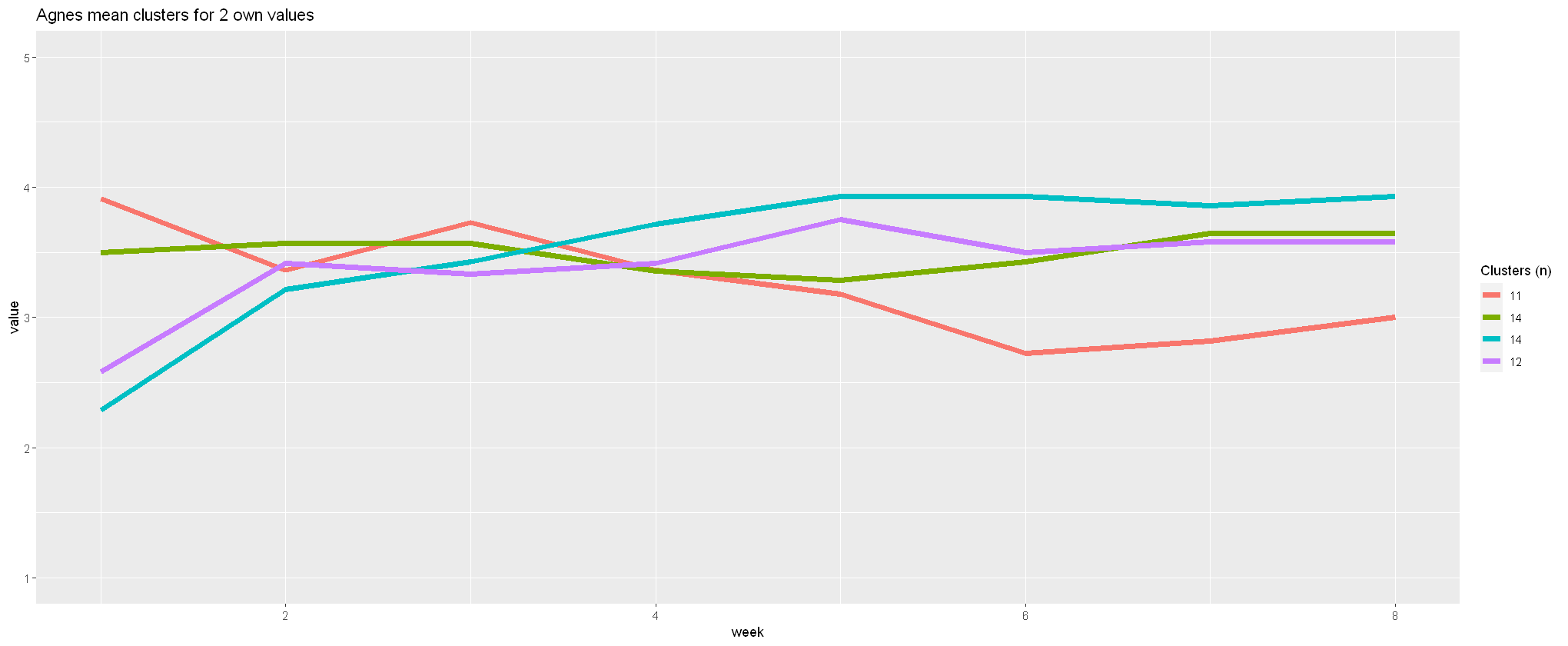


*Supplement G - Average Cluster Time Series for Item 5 (own values)*


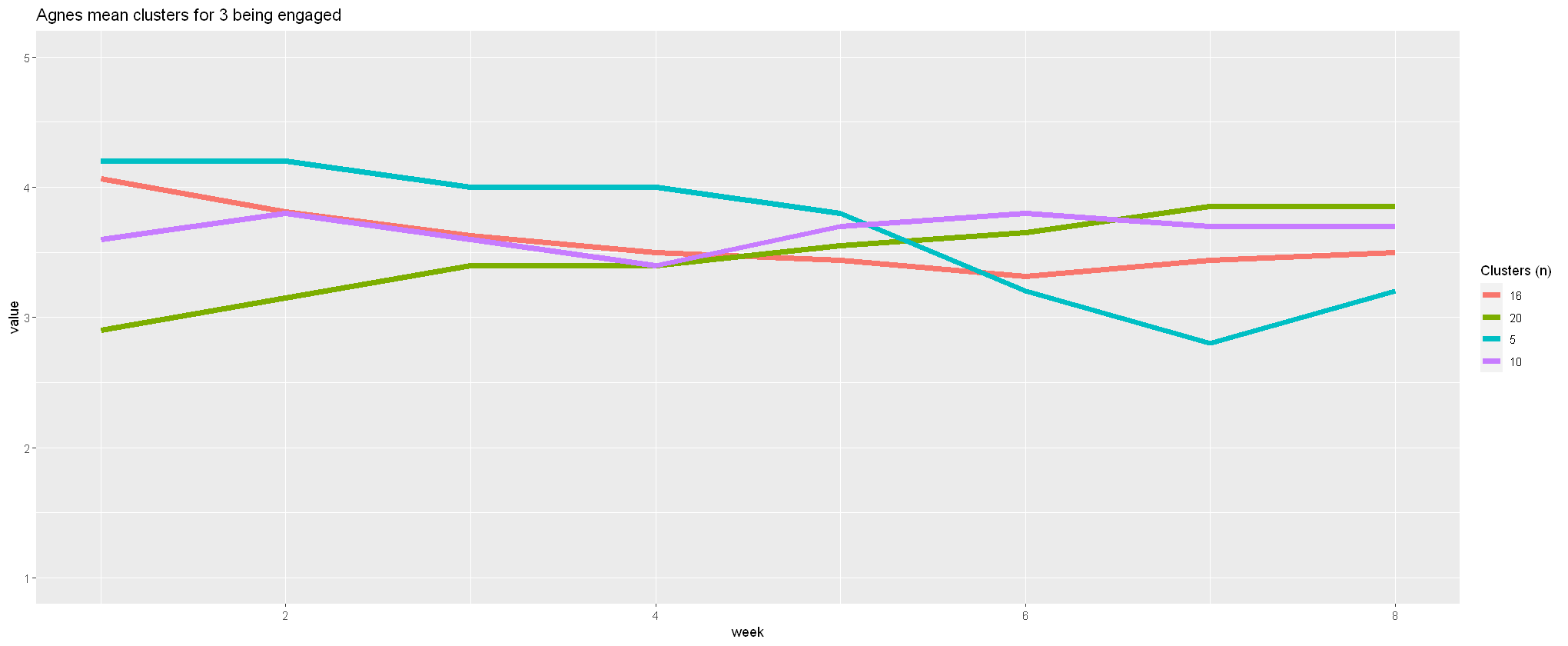


*Supplement G - Average Cluster Time Series for Item 6 (being engaged)*


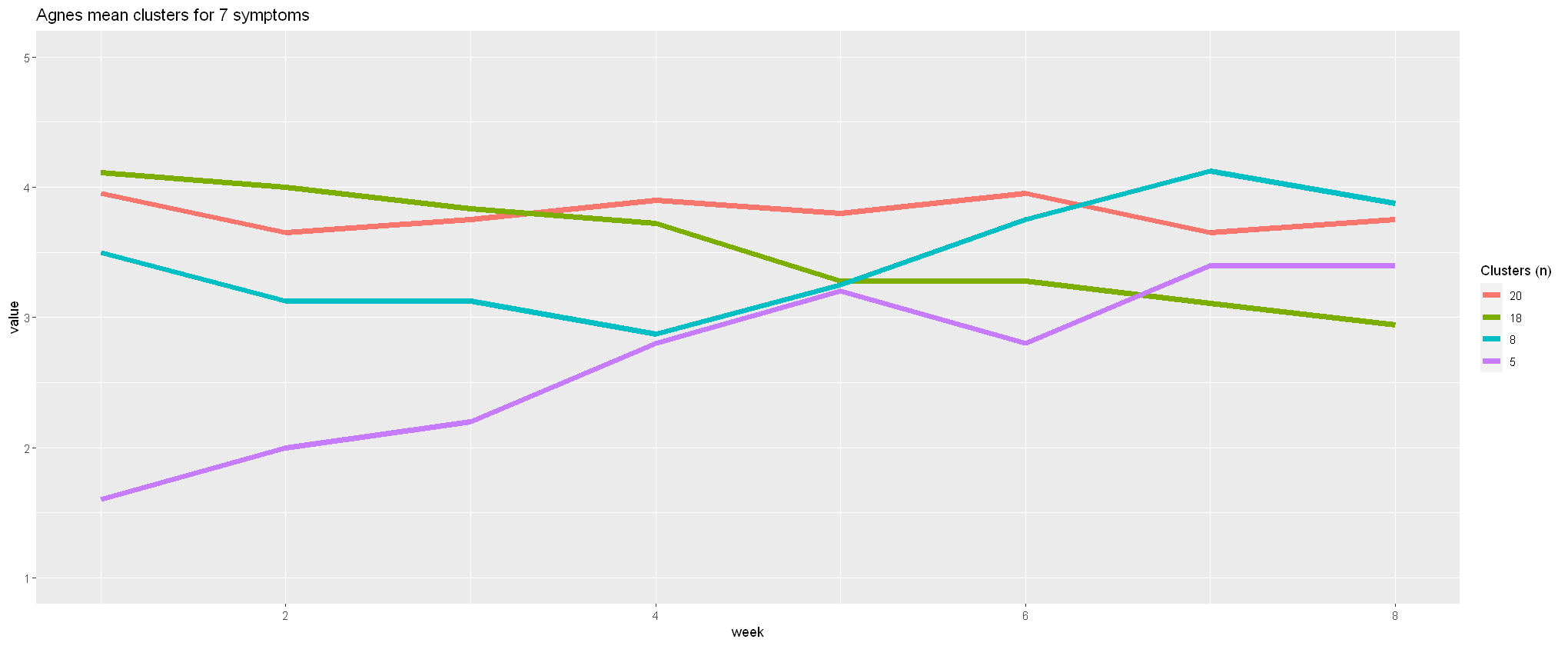


*Supplement G - Average Cluster Time Series for Item 7 (symptoms)*

### Supplement H

To associate the clusters obtained from the AGNES method with well-being at post, linear regression models were calculated. An ANOVA was used to compare the highest mean outcome per group to the lowest mean outcome per group. Even though the small sample size did not lead to expectations of statistical significance, large effect sizes and even some significant differences between the clusters have been found for some items (defusion, acceptance, trending for values). If one, for example, looked at the negative association of group 1 of Item 5 (defusion) with well-being and then went back to the graph that shows the clusters of Item 5 (defusion), one can see that all trajectories included in that clusters are negative, leading to lower levels of defusion capabilities at post than at baseline. We invite the curious reader to make these comparisons for the visualized data for Item 1-7 shown below.


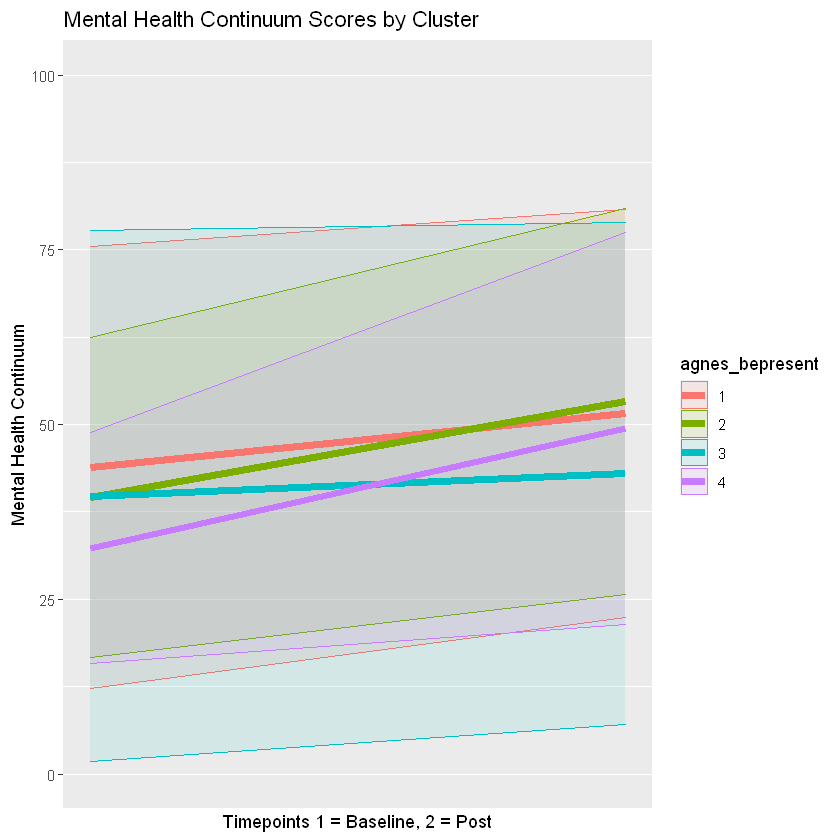


*Supplement H –* *Association Wellbeing with* *AGNES* *Clusters for Item 1 (being present)*


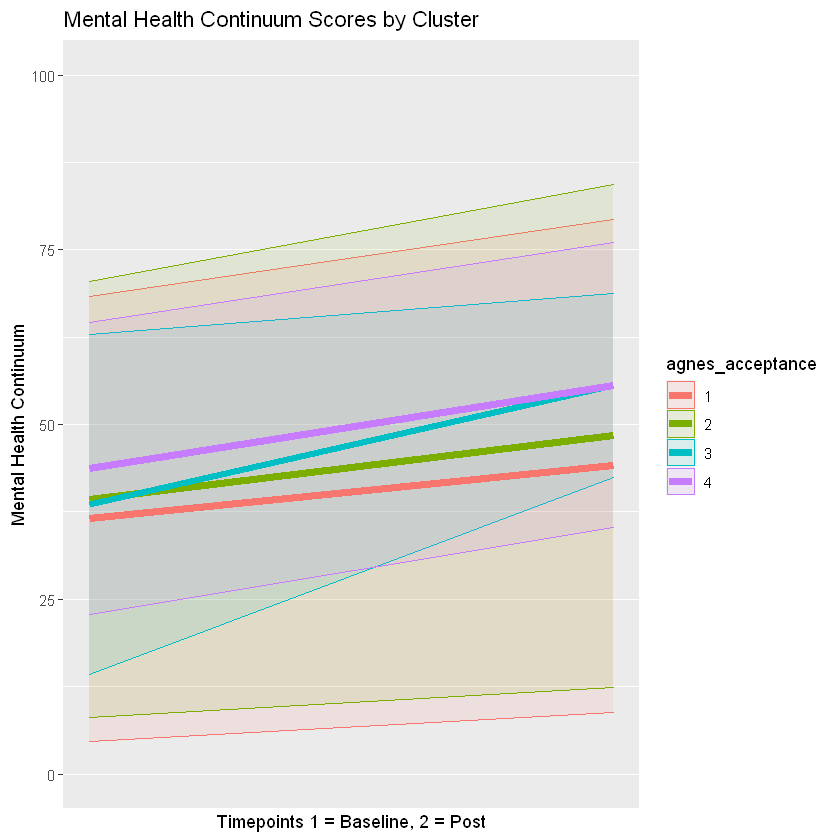


*Supplement H –* *Association Wellbeing with* *AGNES Clusters* *for Item 2 (acceptance)*


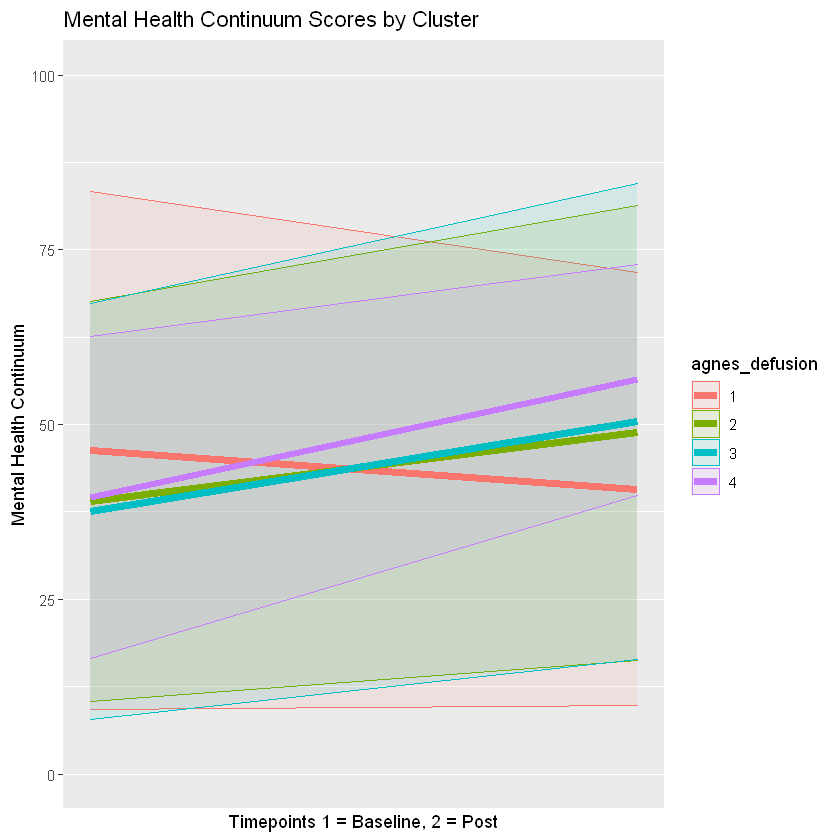


*Supplement H –* *Association Wellbeing with* *AGNES Clusters* *for Item 3 (defusion)*


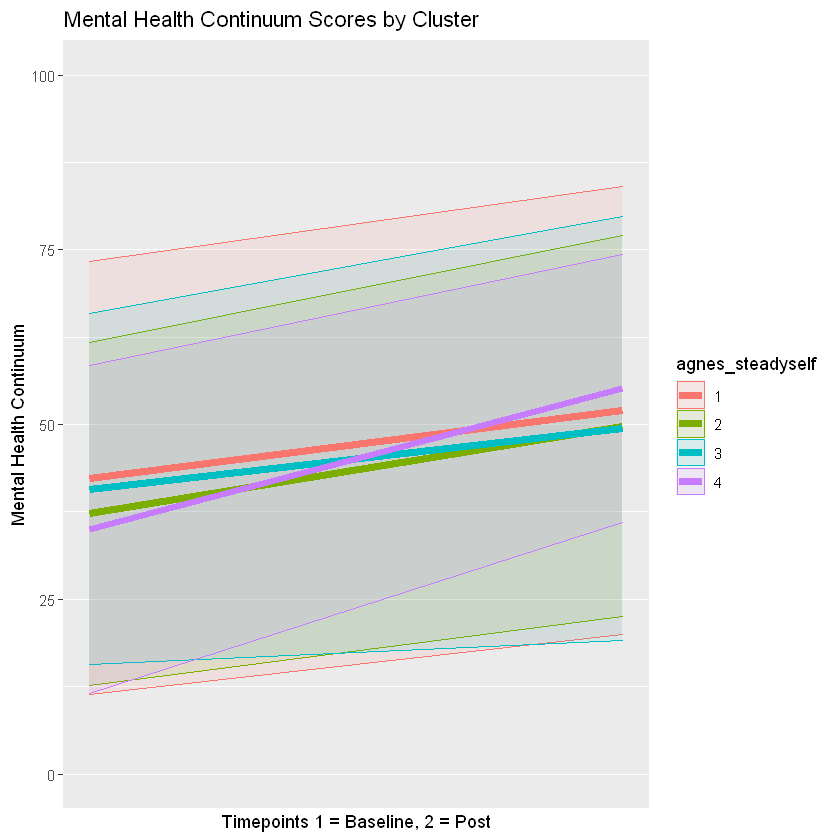


*Supplement H –* *Association Wellbeing with* *AGNES Clusters* *for Item 4 (steady self)*

*
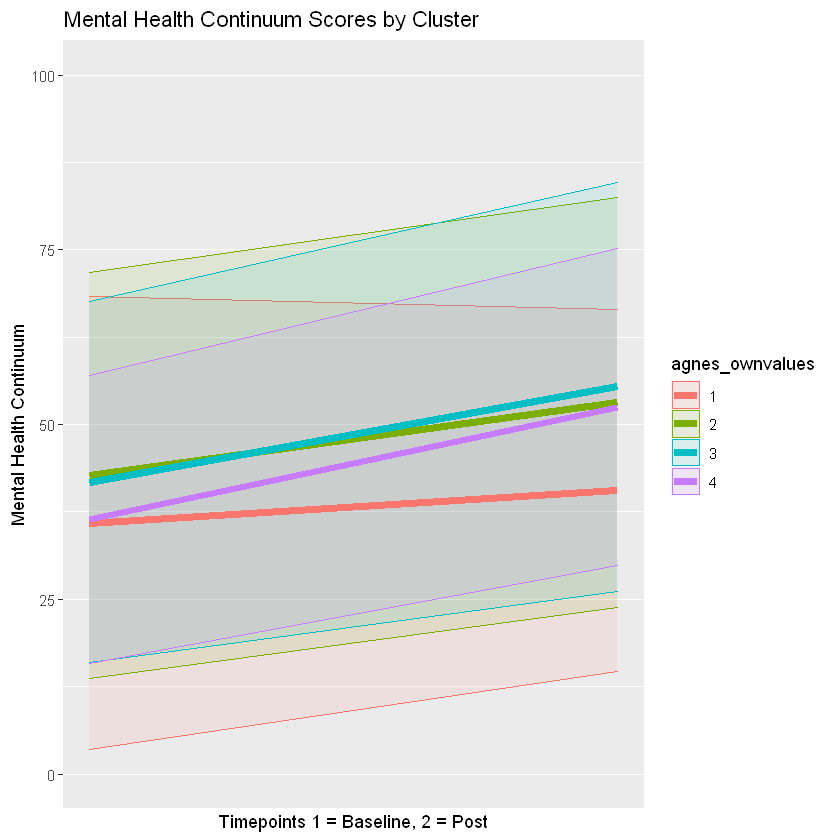
*

*Supplement H –* *Association Wellbeing with* *AGNES Clusters* *for Item 5 (own values)*


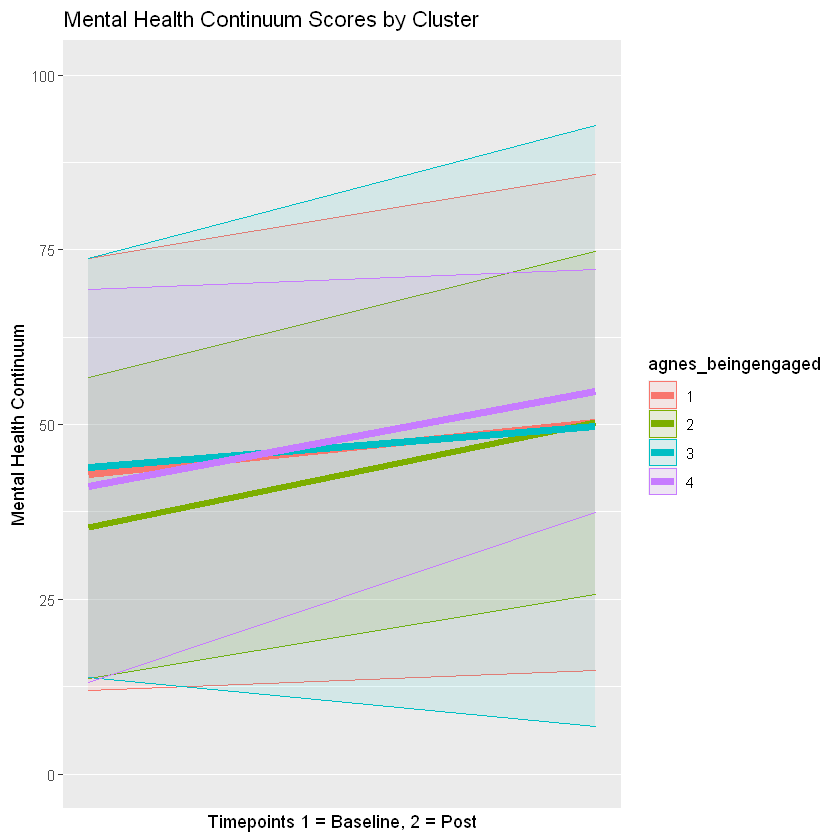


*Supplement H –* *Association Wellbeing with* *AGNES Clusters* *for Item 6 (being engaged)*


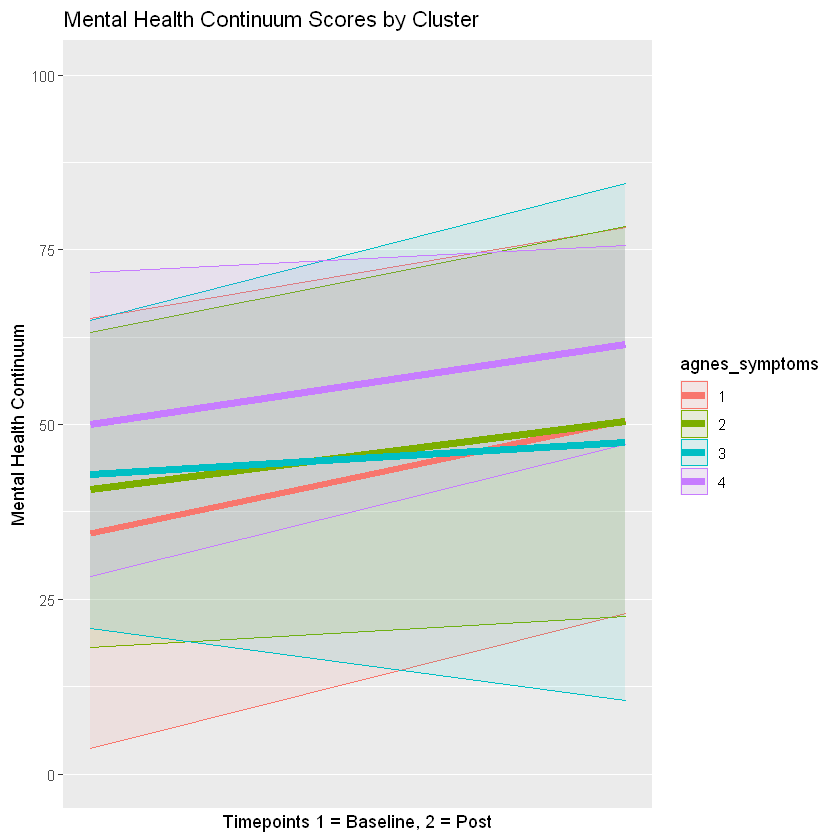


*Supplement H –* *Association Wellbeing with* *AGNES Clusters* *for Item 7 (symptoms)*

Authors: Andrew T. Gloster, Matthias Nadler, Victoria Block, Elisa Haller, Julian Rubel, Charles Benoy, Jeanette Villanueva, Klaus Bader, Marc Walter, Undine Lang, Stefan G. Hofmann, Joseph Ciarrochi, Steven C. Hayes

Correspondence concerning this article should be addressed to Andrew T. Gloster (andrew.gloster@unibas.ch).
